# Autophagy: a double-edged sword in ischemia–reperfusion injury

**DOI:** 10.1186/s11658-025-00713-x

**Published:** 2025-04-07

**Authors:** Lingxuan Tang, Wangzheqi Zhang, Yan Liao, Weijie Wang, Xiaoming Deng, Changli Wang, Wenwen Shi

**Affiliations:** 1 Faculty of Anesthesiology, Changhai Hospital, Naval Medical University, Shanghai, 200433 China; 2 School of Anesthesiology, Naval Medical University, 168 Changhai Road, Shanghai, 200433 China; 3 Basic Medical University, Naval Medical University, Shanghai, 200433 China; 4 School of Nursing, Navy Military Medical University, Shanghai, China

**Keywords:** I/R injury, Autophagy, Mitophagy, Apoptosis, Necroptosis

## Abstract

Ischemia–reperfusion (I/R) injury describes the pathological process wherein tissue damage, initially caused by insufficient blood supply (ischemia), is exacerbated upon the restoration of blood flow (reperfusion). This phenomenon can lead to irreversible tissue damage and is commonly observed in contexts such as cardiac surgery and stroke, where blood supply is temporarily obstructed. During ischemic conditions, the anaerobic metabolism of tissues and organs results in compromised enzyme activity. Subsequent reperfusion exacerbates mitochondrial dysfunction, leading to increased oxidative stress and the accumulation of reactive oxygen species (ROS). This cascade ultimately triggers cell death through mechanisms such as autophagy and mitophagy. Autophagy constitutes a crucial catabolic mechanism within eukaryotic cells, facilitating the degradation and recycling of damaged, aged, or superfluous organelles and proteins via the lysosomal pathway. This process is essential for maintaining cellular homeostasis and adapting to diverse stress conditions. As a cellular self-degradation and clearance mechanism, autophagy exhibits a dualistic function: it can confer protection during the initial phases of cellular injury, yet potentially exacerbate damage in the later stages. This paper aims to elucidate the fundamental mechanisms of autophagy in I/R injury, highlighting its dual role in regulation and its effects on both organ-specific and systemic responses. By comprehending the dual mechanisms of autophagy and their implications for organ function, this study seeks to explore the potential for therapeutic interventions through the modulation of autophagy within clinical settings.

## Introduction

Ischemia–reperfusion (I/R) injury constitutes a pathological cascade initiated by the reestablishment of blood flow to previously hypoxic tissues, presenting a significant challenge to multiple organ systems [1]. It exacerbates morbidity and mortality across a spectrum of diseases, including myocardial infarction, ischemic stroke, acute kidney injury (AKI), trauma, circulatory failure, sickle cell disease, and sleep apnea [2]. Furthermore, it may precipitate pathological responses such as systemic inflammatory response syndrome (SIRS) and multiple organ dysfunction syndrome (MODS) when organ ischemia results in a disequilibrium between metabolic supply and demand [3, 4]. In the context of cardiac function, the reduction in oxygen supply to ischemic tissues is intricately linked to a decline in mitochondrial oxidative phosphorylation, subsequently causing a transition from aerobic to anaerobic metabolism [5]. Consequently, ischemic injury has emerged as a significant challenge in organ transplantation as well as in cardiothoracic, vascular, and general surgical procedures. The objective of reperfusion is to avert cell death induced by ischemia, sustain cellular metabolism, and facilitate the removal of metabolic waste by reinstating the supply of oxygen and nutrients [6]. Nonetheless, it is crucial to acknowledge that the reintroduction of blood flow following prolonged ischemia can potentially exacerbate tissue damage, manifesting as heightened local inflammation, mitochondrial dysfunction, and an acute surge in reactive oxygen species (ROS) production [6, 7] Furthermore, oxidative stress and the initiation of self-damaging tissue responses are also pivotal mechanisms underlying I/R injury [6]. Over recent decades, oxidative stress has garnered significant attention as a pivotal contributor to cell death and tissue damage in I/R injury, particularly in the context of elucidating molecular mechanisms [8]. The reperfusion of blood following ischemia intensifies both specific and nonspecific immune responses within cells, resulting in the release of cytokines and chemokines, inflammation, and excessive apoptosis. These processes collectively trigger a cascade of detrimental cell death pathways, including the activation of autophagy [9].

Autophagy is a crucial cellular cyclic mechanism essential for preserving intracellular homeostasis [10–12]. Functioning as the primary intracellular degradation system, it facilitates the entry of cytoplasmic material into the lysosome via the autophagic pathway, where degradation occurs [3]. The objective of autophagy extends beyond mere material elimination; it operates as a dynamic circulatory system that salvages deteriorating cells and supplies new building blocks and energy for cellular repair and homeostasis by degrading and recycling damaged cellular components [3, 12]. For instance, the inhibition of NOD-like receptor protein 3 (NLRP3) inflammasome activity can enhance the occurrence of mitophagy, consequently mitigating apoptosis during AKI and offering a potential novel target for AKI treatment [5]. Mitophagy generally facilitates cellular adaptation and protection through various mechanisms, including the elimination of damaged mitochondria [13, 14]. In certain instances, excessive autophagy can result in cell death, exemplified by the generation of mitochondrial bursts of ROS following I/R. These cell death modalities encompass necrosis, mitochondrial permeability transition-driven necrosis, ferroptosis, pyroptosis, para-apoptosis, cuproptosis, apoptosis, mitophagy, and autophagy, among others [3, 12]. Therefore, autophagy functions as a double-edged sword, serving both as a mechanism for cellular survival and as a potential pathway to cell death [10].

This review examines the dual role of autophagy in I/R injury, positing that it exhibits both protective and deleterious effects. During ischemic conditions, autophagy mitigates cellular damage and preserves cellular integrity by removing dysfunctional organelles and proteins. Conversely, during reperfusion, autophagy can potentially exacerbate damage and contribute to cell death. Through a comprehensive analysis of autophagy and its regulatory mechanisms in the context of I/R injury, this study underscores the critical importance of understanding the regulatory pathways governing autophagy in I/R injury. We anticipate leveraging its protective effects in therapeutic applications while minimizing potential adverse effects, thereby offering targeted intervention strategies to optimize organ protection and recovery processes.

## The fundamental mechanism of autophagy

Autophagy is an intricate intracellular self-degradation mechanism meticulously governed by a multitude of regulators and signaling pathways, which collectively dictate the initiation, progression, and termination of the process [15]. The most widely recognized form of autophagy involves the extensive processing of cytoplasmic components via the autophagosome-dependent lysosomal pathway, commonly referred to as macroautophagy [16]. In summary, the macroautophagy process comprises several distinct stages: initially, the formation of the phagophore occurs, which is succeeded by the expansion of the autophagosome membrane. This is followed by the fusion of autophagosomes with lysosomes, culminating in the degradation of the sequestered components within the autophagosome [17]. Beyond macroautophagy, the autophagic process also encompasses chaperone-mediated autophagy (CMA) and microautophagy [18]. In the course of the CMA process, the specific degraded protein associates with the chaperone heat shock cognate (HSC) protein 70 via a distinct amino acid sequence known as the KFERQ motif, which facilitates protein degradation within the CMA pathway. This interaction subsequently enables the protein’s translocation into the lysosome through its interaction with lysosome-associated membrane protein (LAMP)2A [18]. In contrast, microautophagy entails the direct engulfment of cytoplasmic components by lysosomes or the invagination of the endoplasmic reticulum [19]. Macroautophagy constitutes a sophisticated intracellular degradation pathway characterized by a multistep mechanism involving numerous critical proteins, which is crucial for preserving cellular homeostasis [20]. The initiation of autophagosome formation in macroautophagy is triggered by the activation of the Unc-51-like autophagy-activating kinase 1 (ULK1) complex, a pivotal component in the autophagy initiation phase, subsequently leading to the development of double-membrane vesicles that encapsulate cellular debris [19]. Adenosine monophosphate (AMP)-activated protein kinase (AMPK) functions as a critical cellular energy sensor and regulator, responding to fluctuations in the intracellular AMP to adenosine triphosphate (ATP) ratio by modulating metabolic pathways to accommodate variations in energy availability. During the initiation of autophagy, AMPK inhibits the mammalian target of rapamycin complex 1 (mTORC1), a principal regulator of cell growth and proliferation, and facilitates the formation of autophagic vesicles by alleviating the inhibition of the ULK1 complex [19, 21]. Subsequently, the ULK1 complex translocates to the endoplasmic reticulum, where phosphatidylinositol 3-kinase (PI3K) III, also referred to as vacuolar protein sorting (Vps) 34, serves as a pivotal enzyme in the regulation of mammalian endocytosis, lysogenesis, autophagy, and intracellular trafficking. Notably, Vps34 is essential during both the initiation and maturation phases of autophagy, facilitating the formation of autophagosomes through the production of phosphatidylinositol 3-phosphate (PI3P). This process recruits autophagy-related proteins such as Beclin-1, WIPI2, and DFCP1ULK1 [22]. The Beclin-1/Vps34 complex facilitates the expansion of autophagic vesicles. This process is initiated by the phosphorylation of B-cell lymphoma 2 (BCL-2) and BCL-2-interacting cell death mediator (BIM) by activated JNK kinases, leading to the release of Beclin-1 and the subsequent dissociation of the Beclin-1/BCL-2 and BIM complexes. The liberated Beclin-1 subsequently activates Vps34, forming a complex that produces PI3P, thereby promoting the elongation of autophagic vesicles [23]. Currently, autophagy-related genes (ATGs) and protein complexes, such as ATG5–ATG12, which involve ATG7, ATG3, and ATG5–ATG12, facilitate autophagy via a ubiquitin-like covalent binding mechanism. This process subsequently enhances ATG8/microtubule-associated protein 1 light chain 3 (LC3) binding, thereby promoting the expansion and closure of autophagosomes [24]. Furthermore, the ATG12–ATG5 complex can associate with ATG16L to form a polymer complex essential for autophagosome assembly [25]. During the elongation phase, the Beclin-1 and PI3K complexes play a crucial role in coordinating the nucleation of the autophagosomal membrane [9]. LC3 plays a pivotal role in the autophagy pathway, wherein it transitions from its cytosolic form (LC3-I) to a membrane-bound form (LC3-II), a process crucial for the formation and maturation of autophagosomes. Initially, the cysteine protease ATG4 cleaves LC3 to generate LC3-I, which is then processed by ATG3, ATG7, and phosphatidylethanolamine to form LC3-II. Following this, LC3-II is amplified, the ESCRT complex facilitates the completion of the closure phase, and LC3-II becomes integrated into the autophagosomal membrane [24]. In the terminal phase, autophagosomes merge with lysosomes to form autophagolysosomes, a process predominantly facilitated by the soluble *N*-ethylmaleimide-sensitive factor attachment protein receptors (SNARE) complex. This complex plays a crucial role in regulating endomembrane fusion events, particularly in the formation of proteins within the secretory pathway and during endocytosis. STX17 (a t-SNARE protein), VAMP8 (a v-SNARE protein), and SNAP29 (a member of the SNAP family) interact to facilitate the trafficking of complexes to the autophagosomal membrane and the subsequent fusion of lysosomes and autophagosomes. This process culminates in the completion of autophagy through the degradation and recycling of cellular components [10, 23]. As discussed above, during this process, the autophagosome recruits lysosomal fusion proteins while the ATG proteins on its outer membrane are sequentially removed. During this process, STX17 undergoes deacetylation, resulting in the embedding of its C-terminal hairpin-like structure within the autophagosome membrane. This structural configuration facilitates interactions with SNAP29 and the HOPS complex, a substantial protein assembly consisting of six core subunits, thereby promoting the fusion of autophagosomes with lysosomes. Consequently, the damaged organelle components are degraded into smaller molecules within the fused autophagosome-lysosome structures and subsequently recycled [10, 22, 26]. To date, macroautophagy has effectively facilitated the lysosomal degradation of target substrates, encompassing protein aggregates, damaged organelles such as mitochondria and peroxisomes, carbohydrates, lipids, nucleic acids, and pathogens [27]. During this process, lysosomes break down complex molecules and release amino acids, fatty acids, and nucleotides, underscoring the significance of autophagy as a mechanism integral to the metabolic precursor cycle [11].

The selective phagocytosis of cytoplasmic material by autophagosomes is governed by highly specific and genetically regulated mechanisms, collectively referred to as selective autophagy; an example of this is the targeted autophagic degradation of mitochondria [16, 24]. Mitochondria, characterized by their double-membrane structure, primarily facilitate ATP production and regulate cellular energy metabolism [28]. Beyond their role as energy producers, mitochondria also participate in diverse physiological processes, including the mediation of Ca^2+^ signaling in most cells [29]. In cardiomyocytes, mitochondria can account for over 30% of the cell volume to satisfy their consistently elevated energy demands. Nonetheless, mitochondria are vulnerable to cellular stressors, including hypoxia, which can result in the generation of ROS and the release of pro-apoptotic proteins. These processes may ultimately culminate in mitochondrial damage and potentially lead to cell death [28]. Mitochondrial quality control represents a critical mechanism in the regulation of mitochondrial size, quantity, morphology, quality, and biological activity [30], playing a pivotal role in sustaining cellular homeostasis and survival. This process encompasses mitochondrial biogenesis, fusion, fission, and mitophagy [25, 28]. In response to cellular stress-induced mitochondrial damage, cells initially preserve their structural integrity and composition through mechanisms including antioxidative defense, DNA repair, protein folding, and degradation [31, 32]. Should the initial defense mechanisms prove inadequate, an extensive quality control system encompassing mitochondrial biogenesis, fusion, fission, and mitophagy is subsequently activated [31, 32]. In instances where damaged mitochondria are irreparable, mitophagy serves as the final defense mechanism to eliminate compromised mitochondria and preserve cellular viability before the onset of apoptosis and necrosis [8]. Thus, the processes of mitochondrial biogenesis, clearance, dynamics, and their interactions collectively form a robust quality control system that responds to pathological stress and sustains mitochondrial function [28]. Currently, mitophagy pathways encompass both the PINK1-Parkin-mediated and the PINK1-Parkin-independent mitochondrial autophagy pathways [33]. These pathways influence the dual role of I/R injury in the precise regulation of the autophagic process [34]. The specific mechanisms underlying these pathways will be elaborated upon in the subsequent sections. The role of gender in autophagy represents a complex and significant area of research. Notably, studies have indicated that female patients with Alzheimer’s disease exhibit a more pronounced accumulation of autophagosomes, autophagic degradation, and mitophagy compared with their male counterparts. This suggests that, owing to greater mitochondrial or protein damage, female patients may engage in compensatory autophagy, potentially accelerating the pathogenesis of Alzheimer’s disease [35]. Furthermore, it is crucial to address not only the differential impact of diseases across genders but also the underrepresentation of women and gender minorities in scientific research. These groups require increased support and opportunities within the scientific community [36]. Further investigation is essential to elucidate the mechanisms driving gender differences in autophagy and to develop personalized therapeutic strategies targeting these pathways.

**Fig. 1:**
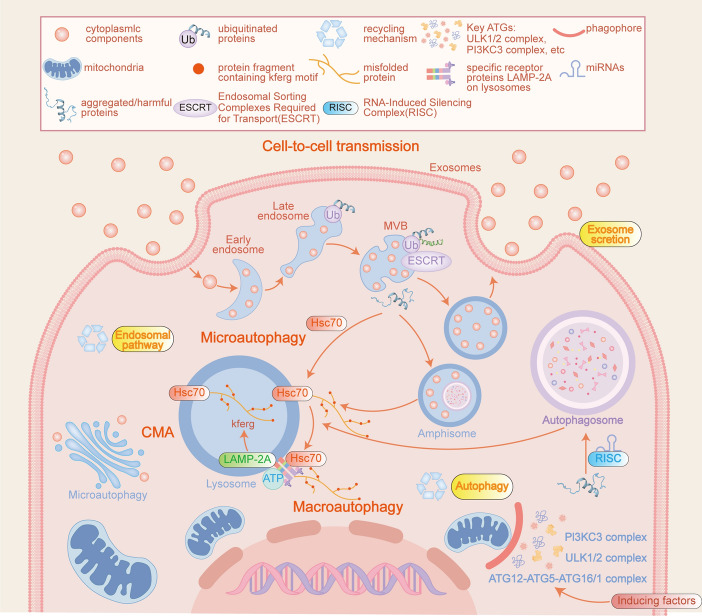
The basic mechanism of autophagy. Autophagy is a cellular process responsible for the degradation and recycling of intracellular components, encompassing three primary forms: macroautophagy, microautophagy, and CMA. Macroautophagy, in particular, serves as an intracellular self-digestion mechanism characterized by a complex sequence of events involving the coordinated action of multiple key proteins. This process can be delineated into four distinct stages: initiation of the autophagosome, elongation of the autophagosome, maturation or blocking of the autophagosome, and the fusion of autophagosomes with lysosomes. (Created using Adobe Illustrator)

Additionally, during ischemia–reperfusion, autophagy may serve as a protective mechanism in early ischemic conditions by eliminating damaged organelles and proteins, thereby preventing the accumulation of toxic substances. However, during reperfusion, excessive autophagic activity may result in the accumulation of undigested materials within autophagic lysosomes, potentially leading to cellular damage. Currently, there exists a significant gap in research concerning the translation of mitochondrial autophagy mechanisms into effective targeted pharmacological interventions. The majority of mitochondrial autophagy inducers currently available are primarily mitochondrial uncoupling agents or mitochondrial toxins, which present numerous limitations. Furthermore, the clinical efficacy of mitophagy modulators remains to be thoroughly validated [37]. A comprehensive understanding of the molecular mechanisms that govern the transition of autophagy from protective to deleterious effects is essential for the development of targeted therapeutic strategies. Such strategies aim to modulate autophagy in the context of I/R injury to promote cellular recovery rather than destruction. A schematic diagram of the basic mechanistic pathway of autophagy is shown in Fig. 1.

## The protective function of autophagy in I/R injury

**Fig. 2:**
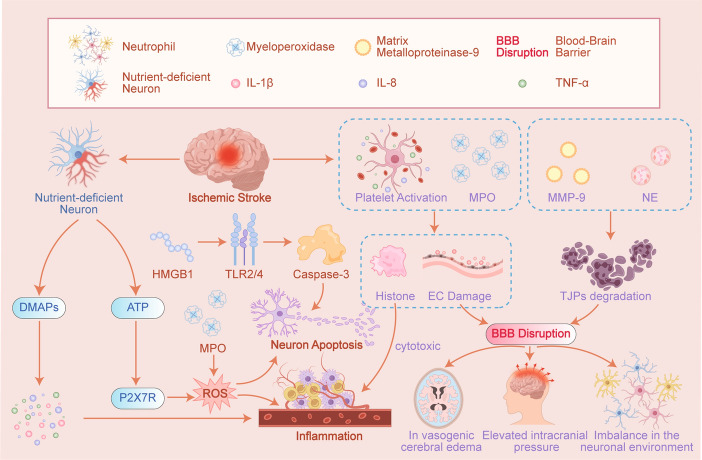
The mechanism of ischemic injury in acute stroke. In the context of acute ischemic stroke (AIS), the obstruction of cerebral blood flow results in damage to cerebral vascular endothelial cells and increased permeability of the blood–brain barrier (BBB). This compromise of the BBB leads to vasogenic cerebral edema, elevated intracranial pressure, and an imbalance in the neuronal microenvironment. Within the framework of AIS, injured neurons may release damage-associated molecular patterns (DAMPs), which subsequently stimulate the production of pro-inflammatory cytokines, including IL-8 and tumor necrosis factor-alpha (TNF-α). The release of cytokines exacerbates cerebral injury by promoting inflammatory responses and neuronal apoptosis. I/R therapy remains a crucial strategy in the management of AIS, as it alleviates both ischemic and reperfusion injuries, thereby protecting cerebral tissue and facilitating neurological recovery. (Created using Adobe Illustrator)

Autophagy potentially serves a protective function in preserving cellular integrity during I/R injury, particularly during the ischemic phase [3, 12]. It functions as an intracellular “cleaner” by eliminating dysfunctional organelles and misfolded proteins, thereby removing debris that could otherwise result in cell death [31]. For instance, during the early stages of tumorigenesis, autophagy contributes to an antitumor response by engaging in oxidative stress management and eliminating dysregulated cells, which helps maintain genomic stability and inhibit tissue damage and inflammation [38]. This clearance mechanism holds significant importance within the central nervous system, as ischemic injury swiftly disturbs the intricate equilibrium of the neuronal environment [33]. During the reperfusion phase, autophagy assumes a crucial role in preserving homeostasis within the intracellular milieu as oxygen and nutrients are reintroduced [10, 11]. It facilitates the recycling of cellular components into usable substrates, thereby supporting ATP production and promoting cellular recovery [20]. This metabolic reorganization represents not merely a response to energy expenditure but also an active strategy that equips cells to address the challenges associated with reperfusion, including oxidative stress and inflammation [27]. Clinical research has demonstrated that intravascular reperfusion therapies, such as intravenous thrombolysis or mechanical thrombus extraction, administered within a defined time window, constitute a relatively safe and restorative intervention for patients experiencing acute ischemic stroke (AIS) [39–41]. Increased mitochondrial fragmentation and fission activity have been documented during the ischemic phase in both brain tissue and cardiomyocytes, as well as throughout I/R injury [42–45]. The neuroprotective effect of autophagy is attributed to its capacity to mitigate the accumulation of toxic proteins and damaged mitochondria, thereby preserving neuronal viability and function [33, 46]. The mechanism of ischemic injury in patients with acute stroke is illustrated in Fig. 2.

The association between mitochondrial injury and the restoration of blood flow represents a critical pathophysiological mechanism following I/R events [47]. Mitochondria that are functionally impaired, along with ROS generated through oxidative stress within these organelles, serve as substrates that can trigger intracellular autophagy [48]. This process facilitates the recovery or degradation of proteins and damaged organelles across various diseases [49]. Mitophagy, a specialized form of autophagy, specifically targets and degrades damaged mitochondria, enabling the recycling of their components [28]. Mitophagy plays a critical role in cellular homeostasis by selectively eliminating and degrading damaged or superfluous mitochondria, thereby preventing the accumulation of mitochondrial DNA mutations and facilitating the reprogramming of cellular metabolism [47]. The PINK1-PRKN/Parkin axis is considered the principal regulator of the PINK1-Parkin-mediated pathway, one of the two mitophagic autophagy pathways discussed above. This pathway initiates selective autophagy by marking damaged mitochondria with ubiquitin chains [50, 51]. In this process, PINK1 is hypothesized to function as a sensor for mitochondrial damage, Parkin as a signal amplifier, and the ubiquitinated chain as a crucial effector for signal transmission [37]. Nevertheless, the ubiquitinated chain does not directly interact with the free autophagic membrane or the associated ATG8 family proteins, indicating that ubiquitinated entities must be anchored to the autophagic membrane via specific molecular pathways [52]. Autophagy aptamers are characterized as proteins possessing mitochondrial ubiquitin-binding domains (UBDs), which facilitate the recognition of ubiquitin tags, as well as LC3 interaction regions (LIRs) that engage with ATG8 family proteins. These proteins include sequestosome 1 (P62/SQSTM1) [24], neighbor of BRCA1 gene 1 (NBR1) [53], nuclear dot protein 52 (NDP52/CALCOCO2), TAX1BP1 [54], and optineurin (OPTN) [51]. As receptors, they function to recognize ubiquitin chains on the mitochondrial surface and also bind to LC3B on phagocytic cell membranes [50]. Conversely, PINK1 may facilitate mitophagy through mechanisms independent of Parkin. For instance, the mitochondrial E3 ubiquitin ligase (MUL1), also known as MITA, can be activated via phosphorylation by PINK1, thereby contributing to the process of mitophagy [55]. Furthermore, the autophagy receptors involved in mitophagy include BNIP3, NIX, FUNDC1, MCL-1, cardiolipin (CL), among others [56]. PINK1 is pivotal in mitophagy, facilitating the removal of damaged mitochondria through the activation of Parkin as well as via Parkin-independent pathways. The process of mitophagy is also associated with the fusion of nascent mitochondria. Optic atrophy 1 (Opa1) is an inner mitochondrial membrane protein crucial for preserving mitochondrial structure and function, and it plays a significant role in regulating mitochondrial fusion and fission [42]. Research indicates that Opa1 undergoes apoptosis-associated modifications, shifting the equilibrium of mitochondrial dynamics toward fission by suppressing fusion [57]. While I/R injury primarily results in mitochondrial dysfunction, causing disruptions in oxidative stress regulation, calcium homeostasis, and apoptosis, targeting mitophagy-related pathways with specific molecules may offer therapeutic benefits for certain patients experiencing ischemic stroke. Certain regulators of mitophagy have demonstrated significant promise in clinical applications, particularly during extended recovery periods, where mitophagy may offer critical neuroprotective benefits and result in improved outcomes [58].

To comprehensively harness the potential of mitophagy in clinical treatment, it is imperative to further investigate and identify therapeutic targets capable of modulating the mitophagy pathway, alongside the development of corresponding pharmacological interventions. By conducting an in-depth analysis of the mechanisms through which autophagy protects cells, we can elucidate the specific pathways involved in mitigating I/R injury. This understanding will establish a theoretical foundation for the development of targeted intervention strategies. Such insights will enhance our ability to artificially sustain cell survival while minimizing the risk of inducing excessive cellular activation. Consequently, this research will offer scientific guidance for the development of therapeutic approaches that effectively harness the protective benefits of autophagy.

## The adverse function of autophagy in I/R injury

Autophagy has been identified as a crucial cellular survival mechanism, facilitating the degradation and recycling of damaged organelles and proteins into nutrients that support cell viability. By regulating the autophagic pathway, cells are effectively maintained during states of homeostasis, stress, and infection. Consequently, autophagy was initially perceived primarily as a self-protective strategy enabling cells to mitigate damage induced by external stimuli [59, 60]. Nevertheless, although autophagy serves as a survival mechanism, its dysregulation may initiate a cascade of events culminating in excessive autophagy and potentially resulting in cell death [33]. The activation process of autophagy is intricate and modulated by environmental factors, with interactions at multiple levels exerting a significant influence on the ultimate outcome [61]. Thus, the protective function of autophagy in I/R injury is not unequivocal [28, 30], and disturbances in intracellular homeostasis beyond a certain threshold of time or magnitude can lead to deleterious effects [62–64]. For instance, in the management of ischemic stroke, while reperfusion strategies such as thrombolysis and thrombectomy are crucial for re-establishing blood flow and enhancing patient outcomes, they may also result in reperfusion injury, which can cause mitochondrial DNA damage and disrupt calcium homeostasis within the cytoplasm and mitochondria [33].

Furthermore, the interplay between autophagy and cell death serves as a critical determinant of cellular fate in I/R injury [10, 11, 65]. The role of autophagy in cell death can be categorized into autophagy-dependent cell death (ADCD or ACD) and autophagy-mediated cell death (AMCD) [10]. The two forms of autophagy associated with cell death are not entirely independent and may coexist within the cell. In certain instances, these modes can intertwine during the process of cell death [66]. ADCD typically occurs when autophagy is excessively activated, and the lysosomal degradation capacity is inadequate to process the substantial number of autophagosomes, which may include endoplasmic reticulum phagocytosis, mitophagy, and self-mutilation. This form of cell death is characterized by the accumulation of autophagosomes, resulting in disruptions to the intracellular environment and damage to organelles. For instance, during reperfusion, the disruption of autophagic flux can result in the accumulation of autophagosomes, thereby creating a toxic intracellular environment that exacerbates cellular damage and may ultimately lead to cell death [7, 10, 11, 20]. This disruption may arise from an imbalance in lysosomal degradation capacity or from the inhibition of autophagosome–lysosome fusion [22]. Lysosomes play a crucial role as degradative organelles within cells, and their proper functioning is essential for maintaining intracellular homeostasis [7, 10, 11]. During I/R injury, the lysosomal degradation capacity may become compromised owing to various factors, including diminished lysosomal enzyme activity and inadequate energy supply resulting from ischemia, both of which impair the enzymatic degradation of substrates [26]. Furthermore, I/R injury may disrupt the intracellular acidic environment, leading to lysosomal acidification disorders that adversely affect lysosomal enzyme activity [33]. The fusion of autophagosomes with lysosomes represents a pivotal stage in the degradation of autophagic substrates [7]. However, during I/R injury, this fusion process may be impeded, leading to the inefficient breakdown of autophagic substrates [26]. AMCD is a distinct form of cell death that is entirely reliant on the autophagic process.

Various forms of cell death include autophagy, necrosis, pyroptosis, apoptosis, and ferroptosis [67]. Autophagy can interact with these cell death processes in complex ways, depending on the specific cellular environment and signaling pathways involved [10]. Biochemically, autophagy is marked by increased expression of autophagy-related proteins such as LC3-II and Beclin-1. In contrast, necrosis is a passive and uncontrolled process triggered by external factors such as physical or chemical damage, leading to cell swelling, membrane rupture, and release of intracellular contents, with biochemical markers including lactate dehydrogenase (LDH) release and increased levels of ROS production [68]. Morphologically, necrotic cells are characterized by organelle swelling and the loss of plasma membrane integrity, whereas apoptotic cells display contraction, nuclear condensation, and DNA fragmentation [69]. Apoptosis is a programmed cell death process initiated by internal or external signals, leading to caspase activation and subsequent cell division and dissolution [70]. Pyroptosis is integral to the immune response, facilitating the elimination of pathogen-infected cells and inducing inflammation to recruit immune cells [71]. In contrast, ferroptosis is distinct from pyroptosis as it is an iron-dependent form of cell death marked by uncontrolled lipid peroxidation within diverse and adaptable mechanisms [9, 63, 72]. Specific autophagic processes, including ferritinophagy, lipophagy, and clockophagy, contribute to the initiation or execution of iron-induced cell death by selectively degrading proteins or organelles that protect against damage [9]. Additionally, other forms of selective autophagy, such as reticulophagy and lysophagy, bolster cellular defenses against damage caused by iron phagophores [7]. For instance, within cardiac tissue, Mammalian sterile 20-like kinase 1 (Mst1), a component of the Hippo signaling pathway [73], exerts a protective influence against cardiac I/R injury. This is achieved through the activation of the Kelch-like ECH-associated protein 1 (Keap1)/nuclear factor erythroid 2-related factor (Nrf2) axis and the suppression of ROS production, indicating a significant role for Mst1 in the transitional management of cardiac I/R injury during heart transplantation [6]. Studies have demonstrated that smoking can exacerbate autophagy through various mechanisms, contributing to cellular aging and tissue damage: free radicals and reactive oxygen species generated by smoking can harm cellular lipids, proteins, and DNA, resulting in oxidative stress; carcinogens present in smoke can damage cellular DNA; and smoking also induces mitochondrial damage and inflammation, which can further impair autophagy [74]. Research has indicated that factors such as stress [75] and environmental pollution [76] may trigger excessive autophagy in the body, potentially leading to depression under chronic stress conditions and severe outcomes like miscarriage in pregnant females. Understanding the mechanism of autophagy in the context of I/R injury is of paramount importance. It is essential to investigate the potential of autophagy as a therapeutic target, ensuring that its activation during treatment aims to preserve tissues and organs rather than exacerbate cellular damage. This understanding is critical for accurately mitigating and regulating the detrimental effects of autophagy in clinical settings, such as organ transplantation.

## The dual action mechanisms of autophagy

In the preceding section, we discussed the enigmatic dual function of autophagy in I/R injury, a process governed by a variety of molecular mechanisms that modulate signaling pathways and influence whether autophagy facilitates cellular survival or leads to cellular destruction following I/R injury [10]. As previously noted, the genes implicated in the regulation of the fundamental processes of this autophagy pathway, collectively referred to as ATGs, encode proteins that are crucial for the formation and maturation of autophagosomes [60, 61]. Previous research has identified that ATGs are integral to processes such as protein secretion, pathogen degradation, and the maintenance of genome stability [16]. The regulation of these ATGs is mediated through both genetic and epigenetic mechanisms, which modulate the intensity and duration of autophagic responses, thereby influencing their protective or harmful outcomes [61].

**Fig. 3:**
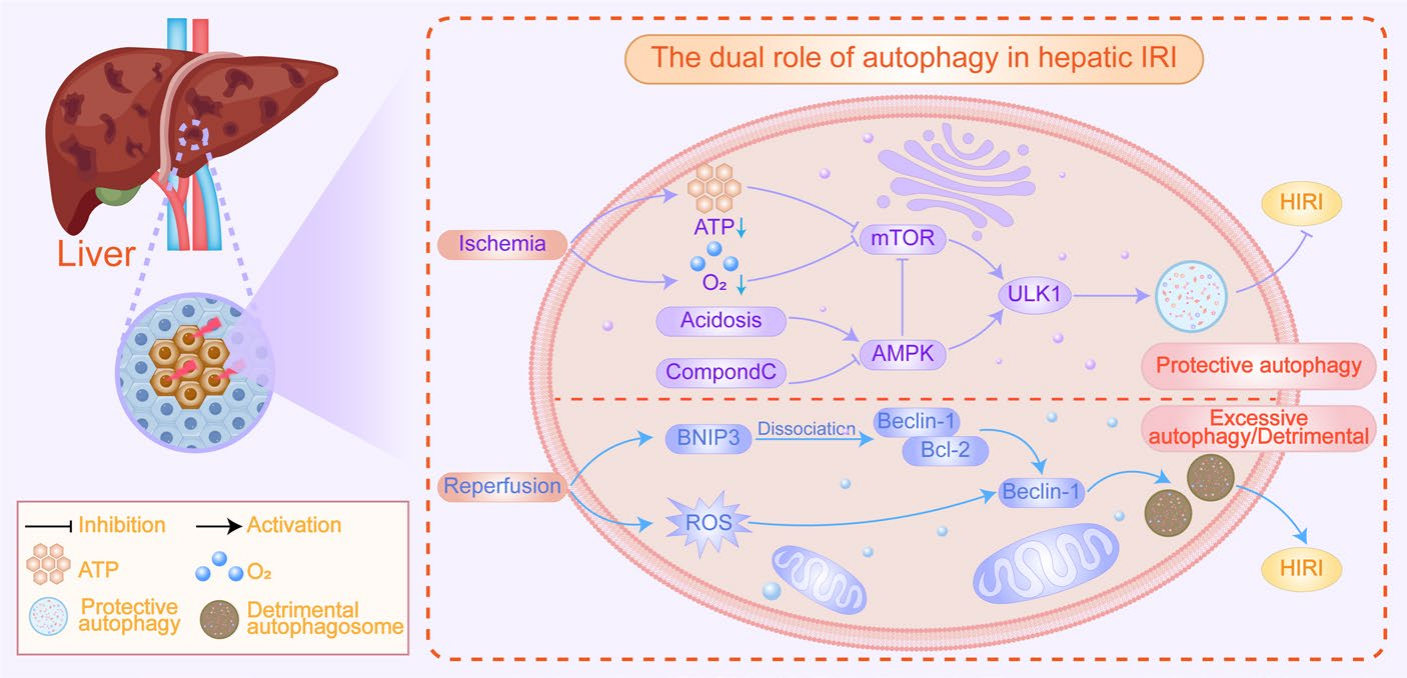
The dual role of autophagy in hepatic IRI. Autophagy plays a dual role in HIRI: moderate autophagy facilitates the clearance of damaged organelles and mitigates oxidative stress, whereas excessive autophagy can exacerbate cellular damage, leading to adverse outcomes. During the ischemic phase, protective autophagy is activated via AMPK stimulation owing to reduced ATP and oxygen levels, which inhibits mTOR, promotes ULK1 activation, and initiates the formation of protective autophagosomes. In the reperfusion phase, increased ROS levels activate BNIP3, resulting in the dissociation of the Beclin-1/Bcl-2 complex and the release of Beclin-1, thereby promoting autophagosome formation and contributing to the clearance of damaged organelles and proteins. However, overactivation of autophagy can lead to the excessive degradation of organelles and proteins, potentially forming harmful autophagosomes and exacerbating cellular damage. Therefore, maintaining autophagic homeostasis is crucial for alleviating hepatic ischemia–reperfusion injury. (Created using Adobe Illustrator)

Furthermore, the extent of autophagy activation is intricately linked to its spatial and temporal dynamics [62]. The thioredoxin-interacting protein (TXNIP)/regulated in development and DNA damage responses 1 (Redd1) complex has been identified as a promoter of autophagosome formation during myocardial I/R, indicating an enhancement in autophagy activation [64]. While TXNIP is known to inhibit autophagosome clearance through the elevation of ROS levels, the formation of autophagosomes induced by TXNIP is not mediated by ROS. This conclusion is supported by evidence showing that the use of ROS scavengers does not impede the increased autophagosome formation observed in hearts overexpressing TXNIP [48, 64]. Ultimately, TXNIP directly interacts with and stabilizes the autophagy regulator Redd1, resulting in mammalian target of rapamycin (mTOR) inhibition and the activation of autophagy. This indicates that increased TXNIP/Redd1 expression represents a novel signaling pathway that exacerbates I/R injury by promoting excessive autophagy during reperfusion [64]. Furthermore, autophagy exhibits dual functions in tumorigenesis, serving as a tumor-suppressing mechanism in the early stages and a cancer-promoting factor in the later stages [77]. The dual mechanisms through which autophagy influences tumor development is illustrated in Fig. 3.

Posttranslational modifications (PTMs) are crucial in the precise regulation of autophagy regulators’ functions [46, 78]. Building on prior research, the mechanism by which the primary amino acid sequence of proteins dictates the material properties of biomolecular condensates is well established [79]. Consequently, PTMs in proteins, including phosphorylation and ubiquitination, function as molecular switches to precisely regulate the dynamics of biomolecular condensates. Ubiquitination, a prevalent and reversible PTM, influences biomolecular assemblies via two primary mechanisms [78, 80]. Firstly, it modifies the molecular structure of protein constituents, thereby altering the physicochemical properties of the biomolecular assemblies. Secondly, it facilitates valence by engaging with binding partners within molecular networks that contain UBDs or ubiquitin-associated domains (UBAs) [80–82]. Furthermore, the accumulation of ubiquitin in individuals with neurodegenerative disorders, including amyotrophic lateral sclerosis (ALS), may be attributed to disruptions in autophagic activity and the homeostasis of stress granules [83]. Consequently, ubiquitination is crucial in regulating the dynamics of biomolecular aggregates associated with these diseases [78]. Drawing from these preclinical studies [78, 80–82], we posit that targeting ubiquitinated biomolecular aggregates holds significant promise in combating diseases such as ALS. PTMs, including phosphorylation and ubiquitination, function as molecular rheostats that dynamically modulate and regulate protein activity, stability, localization, and interactions among proteins such as ULK1, Beclin-1, and PI3K complexes. These processes are crucial in various biological functions, including cellular metabolism, growth, differentiation, and apoptosis [22, 84].

Beclin-1 participates in numerous biological processes, with its most extensively characterized function being its involvement in autophagy. It interacts with and modulates the activity of the PI3KC3/Vps34 lipid kinase, which generates PI3P, a critical regulator in the initiation of autophagy and intracellular membrane trafficking [84, 85]. These interactions can either augment or suppress autophagy, contingent upon the cellular context and the specific signaling pathways activated during I/R injury [22, 62]. For instance, the phosphorylation of ULK1 by AMPK facilitates the initiation of autophagy under energy-depleted conditions [22]. Research indicates that, in intestinal diseases, the expression level of Beclin-1 is frequently downregulated, potentially resulting in a reduction of autophagic activity [60]. Moreover, cyclic GMP-AMP synthetase (cGAS) functions as a DNA-sensing receptor and exhibits increased expression in both human and mouse models of colitis. Research indicates that a deficiency in cGAS can exacerbate colitis and decrease the levels of autophagic proteins, such as Beclin-1 and LC3-II. However, administration of the autophagy activator rapamycin has been shown to significantly alleviate the severity of colitis in cGAS knockout mice [86]. The data indicate that the ubiquitination of Beclin-1 may influence its interaction with anti-apoptotic proteins in the context of human inflammatory bowel disease (IBD) and mouse models of colitis, thereby modulating the equilibrium between autophagy and apoptosis to preserve intestinal epithelial homeostasis. Autophagy induced by ischemia is associated with the activation of AMPK and is suppressed by a dominant negative form of AMPK. However, during reperfusion, autophagy is characterized by an upregulation of Beclin-1 without concurrent AMPK activation [62]. Research indicates that ischemia activates autophagy via AMPK-dependent pathways during cardiac I/R injury, whereas ischemia/reperfusion itself induces autophagy through mechanisms dependent on Beclin-1 but independent of AMPK [21]. Specifically, during myocardial ischemia, myocardial cells experience significant stress due to energy deprivation and ATP depletion, rendering mitochondrial function a critical determinant [87]. Under ischemic and hypoxic conditions, anaerobic glycolysis predominates as the primary metabolic pathway, leading to the accumulation of lactate and hydrogen ions, thereby inducing intracellular acidosis [7]. The resultant decrease in pH and depletion of ATP activate the Na^+^/H^+^ ion exchanger and the Na^+^/HCO_3_^−^ transporter, while inhibiting the Na^+^/K^+^-ATPase, culminating in a substantial intracellular accumulation of sodium ions [31]. The accumulation of sodium ions and alterations in the Na^+^/Ca^2+^ exchanger on the muscle membrane contribute to elevated intracellular calcium levels and mitochondrial swelling [8]. Autophagy is activated as a response to I/R injury, oxidative stress, and energy depletion [8, 48]. Upon the restoration of blood flow and reoxygenation of the respiratory chain, ROS production is intensified, which triggers excessive autophagy and may ultimately result in cell death [48].

Autophagy has been demonstrated to exhibit a dual role in the pathological process of I/R injury [30]. Elucidating the mechanisms by which these molecular regulators operate is essential for comprehending the modulation of autophagy in the context of I/R injury [11, 28]. The biological milieu plays a pivotal role in the execution of autophagy, with its requirements varying across different stages of the autophagic process. Modulating autophagic pathways to alter the mode of cell death post-clinical intervention—transitioning from lethal to protective in the context of cardiovascular disease, or from protective to lethal in cancer therapy—could substantially influence therapeutic outcomes [88, 89]. For instance, in tumor cells, autophagy plays a crucial role in mitigating cytotoxicity by facilitating the removal of deleterious proteins and superfluous or damaged organelles, thereby inhibiting the progression of cellular malignancy. In tumor cells, the reduction in autophagic activity facilitates cellular evasion of apoptosis. Further research is required to substantiate the dual role of autophagy in tumors, which appears to be contingent upon the cellular microenvironment and the level of autophagic activity at specific temporal points [8, 48]. Moreover, the expression patterns of key autophagic molecules across various tumor types and their prognostic implications exhibit variability. To optimize therapeutic outcomes, it is essential to select suitable autophagy inhibitors or agonists on the basis of the specific autophagic and genetic characteristics of the tumor, thereby formulating a targeted and personalized treatment strategy. By focusing on these molecular regulators and their associated pathways, we can potentially augment the beneficial effects of autophagy while minimizing its detrimental impacts. This approach necessitates a comprehensive understanding of the intricate interactions between autophagy and other cellular processes, as well as the development of precise pharmacological tools to modulate these interactions. The primary challenge lies in effectively harnessing these molecular mechanisms to direct autophagy toward cellular rescue rather than cellular destruction.

## The regulation of autophagy in I/R Injury

The regulation of autophagy presents a promising novel strategy for addressing I/R injury; however, its underlying mechanisms are notably complex [28]. The primary molecular regulators of autophagy include mTORC1, AMPK, p53, and endoplasmic reticulum stress (ERS) [7]. Notably, mTOR and Beclin-1, as molecules associated with autophagy, are pivotal during various stages of myocardial ischemia–reperfusion injury (MIRI) [84]. During the ischemic phase, the mTOR operates via the AMPK/mTOR and PI3K/AKT/mTOR signaling pathways [62]. In contrast, the expression of Beclin-1 is upregulated during the reperfusion phase [7]. The regulation of autophagy can be achieved through various strategies, including pharmacological interventions, genetic modifications, and alterations of environmental factors [47, 72, 90].

Regarding pharmacological interventions, the administration of serine/threonine kinase inhibitors, calcium channel inhibitors, and highly selective sodium-glucose cotransporter 2 (SGLT2) inhibitors has been demonstrated to be associated with the self-regulation of autophagy, yielding positive outcomes in clinical treatment [21]. The PI3K/TOR signaling pathway is recognized as a negative regulator of autophagy in mammalian cells. Furthermore, depletion of cellular ATP significantly inhibits mTOR activity without impacting the activation of PI3K or altering intracellular amino acid concentrations [91]. As previously discussed, rapamycin serves as a potent and specific inhibitor of the mTOR pathway, demonstrating its ability to enhance autophagy and exhibiting therapeutic potential in preclinical models of I/R injury [92]. Empagliflozin has been shown to mitigate cardiac microvascular I/R damage through the activation of the AMPKα1/ULK1/FUNDC1/mitochondrial autophagy pathway [21]. In the context of colorectal cancer, excessive activation of the mTOR pathway impedes the initiation of autophagy, thereby facilitating tumor cell proliferation and survival [60].

While genetic manipulation offers precise control over the autophagy process, its application in clinical settings remains constrained. For instance, the inhibition of mTORC1 by circular RNA (circ-FoxO3) to enhance autophagy, or the knockout or overexpression of the ATG gene, exemplifies these limitations [59, 61]. Research indicates that the Sirt1/FoxO3α pathway provides a protective effect by modulating autophagy in hepatic ischemia–reperfusion injury (HIRI), a mechanism that can be disrupted by the specific Sirt1 inhibitor EX-527 [93]. At the molecular level, circular RNAs engage with autophagy-related microRNAs (miRNAs) [94, 95] and proteins [96] to modulate various pathological processes. The interplay between microRNAs (miRNAs) and autophagy is pivotal in the context of neurodegenerative diseases. Both autophagy and miRNAs exhibit dual roles in these diseases [97]. miRNAs are integral to the regulation of autophagy-related genes and signaling pathways, influencing the autophagic process; aberrant miRNA expression can result in autophagic dysfunction, thereby exacerbating the progression of neurodegenerative disorders [98]. Conversely, targeting specific miRNAs offers a therapeutic approach to modulate autophagy levels, potentially mitigating symptoms of neurodegenerative conditions. For instance, inhibiting miR-140, which enhances PINK1-mediated mitophagy, has been shown to alleviate symptoms of Alzheimer’s disease [99]. In the context of spinal cord ischemia–reperfusion injury (SCIRI), noncoding RNAs (ncRNAs) have the capacity to regulate apoptosis, inflammation, autophagy, and oxidative stress, thereby mitigating the effects of SCIRI [100]. The inhibition of the inhibitor Keap1, which regulates the Nrf2—an oxidative stress sensor and pivotal transcription factor for cellular protection against oxidative damage—results in the nuclear accumulation of Nrf2. This accumulation subsequently activates the transcription of genes responsible for encoding a range of cytoprotective, antioxidant, and anti-inflammatory proteins, thereby mitigating oxidative stress damage [6]. For instance, the Mst1 gene has been the subject of extensive research in the context of various reperfusion injuries associated with organ transplantation, including that of the heart [101]. As a pivotal mediator of oxidative stress, this serine/threonine kinase is intricately associated with mitochondrial function and autophagic processes [73]. Exposure to CoCl_2_ has been shown to upregulate Mst1 expression and activate the Keap1/Nrf2 signaling pathway, while exacerbating cellular oxidative damage through Mst1 gene ablation and inhibition of the Keap1/Nrf2 pathway [6]. Furthermore, apoptosis initiated by extrinsic pathways is mediated by transmembrane death receptors, which belong to the tumor necrosis factor receptor (TNFR) family and possess “death domains” [7]. Through the death domain, specific ligands and associated death receptors, such as the apoptosis-stimulating fragment ligand (FasL)/Fas receptor (FasR), TNF-α/TNFR1, TNF-related apoptosis-inducing ligand (TRAIL)/death receptor (DR), and TRAIL/DR5, mediate the transmission of apoptotic signals from the cell surface to intracellular pathways [102]. Additionally, the p53 gene serves as a crucial tumor suppressor, with the p53 protein in the cytoplasm capable of inhibiting the mTOR activity by suppressing AMPK activity [103]. In colorectal cancer, mutations and loss of function in the p53 gene are significantly associated with dysregulated autophagy and tumor progression [60].

Environmental factors are pivotal in investigating the regulatory mechanisms of autophagic activity, as they modulate various signaling pathways and molecular processes [80]. For instance, low-dose hydrogen sulfide (H_2_S) has been shown to mitigate the neuronal damage associated with cerebral ischemia–reperfusion injury (CIRI) [104]. The ubiquitination pathway, along with the PTEN-activated PINK1-Parkin pathway, represents typical regulatory mechanisms operative during mitosis [33, 84]. Furthermore, autophagy fulfills various physiological functions, including the maintenance of cellular homeostasis, promotion of cell survival, regulation of the cell cycle, modulation of oxidative stress, facilitation of muscle regeneration, preservation of the quiescent state of stem cells, and promotion of muscle cell differentiation [105]. In eukaryotic cells, the ubiquitin–proteasome system (UPS) and autophagy constitute two principal cellular degradation pathways that are essential for the clearance of misfolded or unfolded proteins. These pathways are critical for maintaining cellular and tissue homeostasis, preventing alterations associated with aging, and mitigating a range of human diseases [82]. For instance, the inhibition of the UPS results in the compensatory activation of autophagy via multiple mechanisms. Conversely, the suppression of autophagy can either activate or impair the proteasomal pathway, contingent upon the specific cellular context and environmental conditions [51, 83]. Furthermore, components of either system may serve as proteolytic targets for the other [16]. Cells must precisely regulate the induction of autophagy in response to diverse stress conditions. Reversible ubiquitination of the core autophagy-inducing factor, specifically the ULK1 and PI3K complex subunits, has been identified as a universal mechanism for both initiating and terminating autophagy across various cellular contexts [13, 65]. The initiation of autophagy is orchestrated by the ULK1 serine/threonine kinase, which associates with FIP200, ATG13, and ATG101 to form functional complexes [20, 84]. In numerous cellular stress responses, the activation of ULK1 initiates the phosphorylation of downstream factors, subsequently inducing a cascade of autophagy [22]. A key effector of ULK1 is the PI3K-II complex, comprising the lipid kinase Vps34 and the regulatory proteins Beclin-1, Vps15, and ATG14 [22, 84]. ULK1 facilitates the activation of PI3K-III complexes and recruits them to sites of autophagosome formation, where the produced PI3P plays a critical role in the nucleation process of autophagosomes [106]. ATG9, the sole transmembrane protein integral to the core autophagy machinery, is postulated to supply membrane resources essential for autophagosome formation [22]. Furthermore, ATG9 interacts with ATG2 and WIPI proteins (ATG18, a phosphatidylinositol 3-phosphate effector in yeast), playing a crucial role in the initial phases of autophagosome formation originating from the endoplasmic reticulum [65]. Additionally, the ubiquitination process contributes to the timely induction of autophagy through a mechanism of positive feedback [27]. The upregulation of Beclin-1 expression, a pivotal protein involved in the regulation of autophagosome formation and processing, is responsible for the activation of autophagy during reperfusion [84, 85]. In vitro studies have demonstrated that Beclin-1-mediated autophagy is modulated by the BCL-2 protein in cardiomyocytes under conditions of nutrient deprivation, such as amino acid and serum deficiencies [107]. In the human breast cancer cell line MCF-7, Beclin-1 protein expression is markedly reduced and, in certain instances, undetectable [108]. Stable transfection of the Beclin-1 gene has been shown to significantly enhance cellular autophagic activity, thereby reducing carcinogenic potential. Furthermore, ROS may induce Beclin-1-mediated autophagy during reperfusion [84]. In this context, elevated ROS levels serve not only as indicators of an energy crisis but also as critical promoters of autophagy. The overexpression of Beclin-1 resulting from reperfusion is associated with increased oxidative stress [85]. In addition to modulating the expression of Beclin-1, ROS also alter the oxidation state and activity of autophagy-related proteins, thereby facilitating the LC3, which subsequently triggers autophagy [85]. Studies have demonstrated that Beclin-1 inhibits tumorigenesis by inducing autophagic cell death in tumor cells. However, the downregulation of Beclin-1 gene expression markedly diminishes the autophagic response, shielding tumor cells from autophagic cell death and consequently promoting their continued proliferation [109]. Receptor-mediated mitophagy, encompassing the BNIP3 and FUNDC1 pathways, facilitates the recruitment of damaged mitochondria to autophagosomes through the binding to specific proteins [110]. In addition to the role of autophagy in I/R, mitophagy is critically involved as a mechanism of cell death. An analysis of the expanding significance of mitophagy in various other diseases is presented in Table 1.

**Table 1:** Effect of mitophagy in diseases

Disease name	Clinical manifestation	Mechanism	Protective effects	Harmful effects	Refs.
Neurodegenerative diseases	Alzheimer’s disease	Alzheimer’s disease (AD) is attributed to neuronal and synaptic dysfunction resulting from the aberrant deposition of β-amyloid (Aβ) and the accumulation of hyperphosphorylated Tau protein (pTau). The restoration of mitophagy plays a crucial role in inhibiting Aβ formation, reducing its accumulation, and mitigating the hyperphosphorylation of pTau, thereby contributing to the prevention of cognitive dysfunction	Mitophagy plays a crucial role in the clearance of impaired mitochondria by identifying and removing dysfunctional organelles, thus reducing oxidative stress and cellular damage in neuronal cells. By preserving mitochondrial mass, autophagy maintains the cellular energy supply, thereby enhancing neuronal cell viability. Furthermore, the removal of damaged mitochondria can suppress the release of inflammatory mediators, thereby diminishing neuroinflammation and contributing to the maintenance of cerebral homeostasis	Accelerated neuronal degeneration: Dysregulation of the mitophagy pathway can lead to the accumulation of mitochondrial damage, thereby accelerating neuronal degeneration Promotion of amyloid formation: Impairments in autophagic processes may affect the metabolism of amyloid precursor proteins, consequently facilitating the formation of Aβ plaques, which are a primary pathological hallmark of Alzheimer’s disease Increased abnormal phosphorylation of Tau: Mitochondrial dysfunction may contribute to aberrant phosphorylation of the Tau protein, resulting in the formation of neurofibrillary tangles, another key pathological feature	[111–114]
	Parkinson’s disease	Parkinson’s disease (PD) is attributed to the degeneration of dopaminergic neurons within the substantia nigra, with its pathogenesis closely associated with mutations in critical proteins, including α-synuclein (α-syn), leucine-rich repeat kinase 2 (LRRK2), and vacuolar protein sorting 35 (VPS35). These mutations influence disease progression by modulating the process of mitophagy	Ensure mitochondrial quality control: Mitophagy plays a crucial role in preserving the health of the mitochondrial population, thereby ensuring a consistent energy supply and normal cellular metabolism Inhibition of apoptosis: The G2019S mutation in LRRK2 has been shown to impede the removal of Miro from the outer mitochondrial membrane (OMM), consequently delaying mitochondrial arrest and the process of mitophagy	Facilitates α-synuclein aggregation: The overexpression of α-synuclein results in the activation of p38 mitogen-activated protein kinase (p38MAPK), which subsequently induces mitochondrial dysfunction and neuronal apoptosis Exacerbated neuroinflammation: Impaired mitophagy may trigger a neuroinflammatory response, further compromising neuronal integrity and exacerbating the pathogenesis of Parkinson’s disease	[115–119]
	Huntington’s disease	In Huntington’s disease (HD), the aberrant expansion of the huntingtin protein (Htt) gene sequence impairs the initiation of mitophagy and the recruitment of the LC3 protein to mitochondria. This disruption results in the accumulation of damaged mitochondria, thereby contributing to the progression of the disease	Preservation of mitochondrial function and quality control: Mitophagy plays a crucial role in sustaining mitochondrial function and ensuring quality control in Huntington’s disease, a neurodegenerative disorder characterized by impaired mitochondrial energy production and disrupted biogenesis and quality control processes. This mechanism potentially offers neuroprotection Elimination of aberrant proteins: A hallmark of Huntington’s disease pathology is the accumulation of mutant huntingtin protein aggregates within neurons. Mitophagy may facilitate the removal of these aberrant proteins, thereby mitigating neuronal damage	Mitochondrial dysfunction: As a class II member of ATPase, valosin-containing protein (VCP) has the capacity to bind to the mutant Htt, resulting in its accumulation within the mitochondria. This process triggers an overactivation of mitophagy via the PINK1-Parkin pathway, which culminates in neuronal death Oxidative stress and neuronal death: Impairments in mitophagy can induce oxidative stress, thereby contributing to neuronal death and exacerbating the pathogenesis of Huntington’s disease	[120–122]
	Amyotrophic lateral sclerosis	Amyotrophic lateral sclerosis (ALS) is attributed to mutations in multiple genes, leading to the accumulation of damaged mitochondria at the distal ends of axons, thereby diminishing neuronal survival	In the context of ALS, mitophagy plays a crucial role in preserving mitochondrial function and ensuring cellular homeostasis by facilitating the removal of damaged mitochondria	Mitophagy dysfunction: The gene encoding copper/zinc superoxide dismutase, specifically the mutant form SOD1, impedes retrograde mitochondrial transport in neurons	[123, 124]
Cardiovascular diseases	Cardiomyopathies	Excessive activation of mitophagy results in the extensive sequestration of mitochondria, thereby disrupting the energy supply to cardiomyocytes and culminating in cellular damage	Myocardial protection involves the regulation of mitophagy in cardiomyocytes under both physiological and stress conditions, which contributes to cardiomyocyte protection. Mitophagy facilitates the degradation and removal of damaged or dysfunctional mitochondria, thereby preserving mitochondrial quality and quantity balance within the cell and maintaining cellular homeostasis	Mitophagic dysfunction is characterized by the ablation of dynein-associated protein 1 (Drp1), which inhibits mitochondrial fission, markedly elevates Parkin levels, and augments Parkin-mediated mitophagy. This overactivation results in mitochondrial depletion and culminates in severe cardiomyopathy	[125, 126]
	Heart failure	Under conditions of sustained stress, the mitochondria within cardiac muscle cells are susceptible to damage. Should mitophagy be inadequate in removing the impaired mitochondria promptly, this can initiate apoptosis in cardiomyocytes, potentially culminating in heart failure (HF). Moreover, the risk of heart failure is markedly elevated when the heart experiences prolonged abnormal hemodynamic pressure overload	Enhancement of cardiac function: Pharmacological interventions, notably mTOR inhibitors such as rapamycin, can facilitate mitophagy, which is instrumental in preserving the integrity and functionality of the mitochondrial network by eliminating damaged mitochondria. This process consequently enhances the overall function of the heart	Mitophagy disorder: Under conditions of cellular stress, the activation of the mitochondrial protease OMA1 facilitates the cleavage of Opa1 from its long isoform (L-Opa1) to its short isoform (S-Opa1), thereby inhibiting mitochondrial fusion and promoting mitochondrial fragmentation. This process ultimately results in cellular necrosis, fibrosis, and ventricular remodeling	[127–129]
	Myocardium aging	The functionality of cardiomyocytes is constrained by the aging process, wherein mitophagy serves a crucial role in the elimination of dysfunctional mitochondria and the mitigation of age-associated pathologies	Mitigate cardiac aging: Parkin has the potential to retard the cardiac aging process through the induction of ubiquitination of the K63 chain of TBK1, which subsequently activates the Parkin–TBK1–P62 signaling pathway, thereby promoting mitochondrial autophagy	Reduced mitophagy: The accumulation of PINK1 and Parkin within mitochondria results in diminished mitochondrial ubiquitination during cellular senescence, thereby leading to decreased mitophagy and subsequently heightened cellular damage and senescence	[88, 130]
Immunity	Innate immunity	The inflammasome is a multiprotein complex formed by intracytoplasmic pattern recognition receptors. Among these, the NLRP3 inflammasome plays a crucial role in the non-specific recognition mechanisms of innate immunity, significantly influencing the immune response and the pathogenesis of various diseases. Furthermore, mitophagy serves as a negative regulator of NLRP3 inflammasome activation by facilitating the timely removal of damaged mitochondria	Mitophagy plays a crucial role in modulating inflammatory responses by regulating the production of inflammatory mediators. It achieves this by degrading pro-inflammatory mitochondrial DNA and diminishing the release of inflammatory signals, thereby mitigating excessive inflammatory reactions. Furthermore, mitophagy contributes to the survival and energy maintenance of immune cells, particularly during pathogenic infections or inflammatory conditions. Additionally, mitophagy enhances the functionality of antigen-presenting cells, such as dendritic cells, thereby augmenting the immune response to pathogens	Mitochondrial metabolic disorders and the regulation of macrophage phenotypes are critical areas of study within immunology. Macrophages, which are pivotal components of the innate immune system, can be classified into classically activated (M1) and alternatively activated (M2) phenotypes, on the basis of their activation states and functional roles. The metabolic demands of M1 and M2 macrophages differ significantly, with mitochondrial metabolism playing a crucial role in the phenotypic transition between M1 and M2 states. Furthermore, certain pathogens may exploit host cell mitophagic pathways to enhance their survival and replication, thereby complicating host–pathogen interactions	[131–134]
	Adaptive immunity	During viral infections, there is a temporary reduction in the mitotic activity of CD8 + T cells and natural killer (NK) cells, accompanied by an accumulation of depolarized mitochondria. This is followed by an upregulation of mitophagy, a process that efficiently eliminates reactive oxygen species (ROS) and facilitates mitochondrial depolarization. This sequence of events subsequently induces the formation of memory in NK cells post-infection	Mitophagy plays a crucial role in sustaining the normal function and metabolic activity of immune cells, including T cells and B cells, by facilitating the removal of damaged mitochondria. This process is essential for the efficient execution of immune responses. Furthermore, during the activation of immune cells, mitophagy can inhibit mitochondria-dependent apoptosis, thereby enhancing the survival and proliferation of these cells	Abnormal mitophagy can result in impaired immune cell function, which adversely affects the efficiency and precision of immune responses, consequently diminishing the body’s immune defenses. This impairment may also lead to a reduction in the number of immune cells, thereby compromising the overall functionality of the immune system. Furthermore, dysregulation of mitophagy may intensify the inflammatory response, potentially inducing a chronic inflammatory state that can inflict damage on the body	[135, 136]
Metabolic syndrome	Obesity	Severe obesity disrupts metabolic pathways, including those involving glucose and lipids, thereby impairing normal mitochondrial metabolism and contributing to the development of metabolic syndrome	Autophagy preserves mitochondrial function and cellular health by ensuring the efficient removal and recycling of damaged mitochondria and regulating the biogenesis of new mitochondria. This process contributes to the prevention of age-related obesity and chronic diseases	Mitophagy influences lipid metabolism by modulating the functionality of catabolic brown adipose tissue (BAT) and facilitating the differentiation of white adipocytes	[137, 138]
	Diabetes mellitus	Impaired mitophagy in white adipose tissue (WAT) results in excessive ROS production, subsequently inducing oxidative stress-mediated activation of the mitogen-activated protein kinase (MAPK) pathway. This activation disrupts insulin signaling, thereby contributing to insulin resistance in other insulin-responsive organs	Protects pancreatic islet cells from inflammation: Mitophagy prevents inflammation-induced damage to cells in diabetes	The development of type 2 diabetes is associated with impaired mitochondrial autophagy, which facilitates the onset of insulin resistance and contributes to the progression of type 2 diabetes	[139–141]
Cancer	Cancer cells	In an aerobic environment, cancer cells exhibit a preference for energy production via glycolysis over mitochondrial oxidative phosphorylation. The influence of mitochondrial autophagy-related proteins facilitates the reduction of the mitochondrial network and enhances the conversion of glucose to lactate through the glycolytic pathway, thereby satisfying the energy requirements of cancer cells. Additionally, in numerous tumor cells, the loss or functional impairment of the Parkin gene is prevalent, leading to defective mitochondrial autophagy and contributing to tumor progression	Mitophagy plays a critical role in tumor progression by modulating cancer development, impacting metabolic plasticity, stem cell characteristics, and the tumor microenvironment. Additionally, mitophagy predominantly facilitates cell survival, particularly under stress conditions induced by cancer therapies	Mitophagy abnormalities can influence the effectiveness of tumor therapies, potentially contributing to drug resistance and impacting overall cancer treatment outcomes. In certain instances, excessive mitophagy may result in cell death. Furthermore, the dysregulation of mitophagy might be implicated in the progression of tumors	[34, 142, 143]
	Cancer stem cells	Cancer stem cells are characterized by their capacity for self-renewal, differentiation, proliferation, and metastasis. The perinuclear localization of mitochondria, elevated membrane potential, reduced mitochondrial DNA (mtDNA) content, decreased intracellular ROS concentration, and diminished oxygen and glucose consumption in cancer stem cells contribute to their enhanced ability to maintain a quiescent state	Mitophagy is integral to the maintenance of stem cell health and regeneration, as it facilitates the removal of damaged or surplus mitochondria. This process is crucial for the self-renewal and differentiation of stem cells, encompassing both induced pluripotent stem cells (iPSCs) and cancer stem cells (CSCs)	Mitophagy significantly contributes to the metabolic reprogramming of cancer stem cells, potentially serving as a critical determinant in tumor progression. Furthermore, impairments in mitophagy may be linked to the survival and proliferation of cancer stem cells, thereby influencing tumor development and therapeutic outcomes	[144, 145]
Skeletal muscle aging	Muscular dystrophy	Skeletal muscle serves as the dynamic core of the human locomotor system. During the initial phases of myogenic differentiation, DRP1-mediated mitochondrial fission works in concert with mitophagy to facilitate the removal of damaged mitochondria. Following this, the activation of peroxisome proliferator-activated receptor gamma coactivator-1 alpha (PGC-1α) enhances mitochondrial biogenesis, leading to the formation of new mitochondrial networks that fulfill the energy demands associated with skeletal muscle development	Facilitates muscle cell regeneration: Mitophagy plays a crucial role in the regeneration of muscle cells following injury. By eliminating dysfunctional mitochondria and enhancing the production of optimally functioning mitochondria, it supports the process of muscle regeneration and contributes to functional recovery	Mitochondrial dynamic imbalance and autophagy dysfunction: An imbalance in mitochondrial dynamics, coupled with impaired mitochondrial autophagy, can result in varying degrees of muscle atrophy. In such cases, mitochondrial dysfunction adversely impacts muscle health and functionality Muscle damage and functional decline: Aberrations in mitophagy may precipitate muscle damage and functional decline, thereby compromising muscle health and overall athletic performance	[146, 147]
Kidney diseases	Acute kidney diseases	The effect of mitophagy on acute kidney injury (AKI) is twofold; for example, mTOR inhibitors can enhance mitophagy by inhibiting mTOR, thereby reducing renal tubular cell damage, but inhibiting mTOR will promote apoptosis, inhibit renal tubular cell proliferation, and is not conducive to the recovery of AKI	Mitophagy plays a crucial role in mitigating oxidative stress by facilitating the removal of damaged mitochondria, thereby decreasing the accumulation of ROS generated by these organelles. This process effectively diminishes oxidative stress-induced damage to renal tubular cells. Additionally, mitophagy may contribute to the repair and regeneration of impaired tubular cells, thereby promoting the restoration of kidney function	Mitochondrial dysfunction: An imbalance in mitophagy, whether excessive or insufficient, can result in mitochondrial dysfunction, thereby intensifying damage to renal tubular cells Exacerbated inflammatory response: Aberrant mitophagy may facilitate the release of inflammatory mediators, thereby amplifying the inflammatory response in the kidneys and consequently aggravating acute kidney injury (AKI)	[5, 148]
	Chronic kidney diseases	Chronic kidney disease (CKD) is characterized by a progressive decline in renal function and an intensification of renal fibrosis. Throughout the progression of CKD, the accumulation of damaged mitochondria induces an oxidative stress response, which further aggravates apoptosis in renal tubular cells and exacerbates kidney damage. Consequently, the removal of superfluous mitochondria is crucial for preserving the homeostasis of the intracellular environment in renal cells	Mitophagy plays a crucial role in preserving mitochondrial homeostasis, serving as a significant mechanism that is often nephroprotective. It can decelerate the progression of renal fibrosis and contribute to the protection of kidney cells from damage and apoptosis	Exacerbation of renal injury is characterized by impaired mitochondrial function, leading to an excessive generation of ROS. This overproduction of ROS induces oxidative stress, thereby contributing to further deterioration of renal tissue. Moreover, dysfunctional mitochondria release signaling molecules that not only inflict cellular damage but also activate inflammatory pathways	[149, 150]
Liver diseases	Alcoholic liver disease	The liver serves as the primary site for alcohol metabolism, and excessive or prolonged alcohol exposure can result in compromised mitochondrial function within liver cells, ultimately leading to hepatic cellular damage. Effective prevention of alcohol-induced liver damage can be achieved through the removal of damaged and abnormal mitochondria	Mitophagy facilitates the elimination of damaged or dysfunctional mitochondria induced by alcohol consumption, thereby safeguarding hepatic cells from additional harm	Mitochondrial dysfunction: Aberrations in mitophagy can result in mitochondrial dysfunction, thereby accelerating the progression of alcoholic liver disease (ALD). Alcohol consumption has been shown to induce mitochondrial damage, and the dysregulation of mitophagy may further intensify this deleterious process	[151, 152]
	Nonalcoholic fatty liver disease	Nonalcoholic fatty liver disease (NAFLD) represents a metabolic syndrome with an etiology that remains incompletely understood. Mitophagy plays a crucial role in sustaining normal metabolic processes and lipid clearance. Conversely, the aberrant accumulation of lipids in the liver indicates a potential dysfunction in mitophagy	Mitophagy plays a crucial role in maintaining mitochondrial homeostasis and function by mitigating hepatic steatosis and the progression of NAFLD through the clearance of damaged or dysfunctional mitochondria and the activation of mitophagic pathways. Furthermore, mitophagy contributes to cellular health by facilitating the removal and recycling of impaired mitochondria and regulating the biogenesis of new mitochondria. This process is essential for sustaining cellular health and preventing the onset of age-related chronic diseases	Mitochondrial dysfunction: Prolonged consumption of a high-fat diet may result in decreased mRNA and protein expression levels of PINK1-Parkin, consequently contributing to lipid accumulation and the development of nonalcoholic steatohepatitis	[153, 154]
Lung diseases	Acute lung injury	In the context of acute lung injury (ALI), mitophagic activity is upregulated with the objective of removing damaged mitochondria. While this process can disrupt mitochondrial homeostasis and potentially result in excessive mitochondrial clearance, the augmentation of mitophagy contributes to the maintenance of mitochondrial equilibrium, thereby serving a protective function for lung tissue in ALI	Mitigate mitochondrial damage: In the context of ALI, alveolar epithelial cells and endothelial cells experience oxidative stress and inflammatory damage. Mitophagy plays a crucial role in eliminating damaged mitochondria and reducing the production of mitochondrial ROS, thereby minimizing cellular damage Preserve cellular viability: Reduced expression of Parkin diminishes mitophagy, facilitates mitochondrial fusion and repair, and prevents excessive mitophagy from eliminating surplus mitochondria	Cellular damage resulting from excessive autophagy: In the context of ALI, the overactivation of mitophagy can result in a reduction in mitochondrial quantity, thereby impairing cellular energy metabolism and viability Mitochondrial dysfunction: The activation and upregulation of the PINK1-Parkin-mediated mitophagy pathway facilitate the removal of surplus mitochondria and contribute to elevated levels of apoptosis	[155–157]
	Chronic obstructive pulmonary disease	In chronic obstructive pulmonary disease (COPD), the regulation of mitochondrial quality is essential for maintaining lung cell homeostasis, given the substantial energy requirements and the crucial reliance on mitochondrial function	Mitigating cellular damage: Mitophagy may attenuate cellular damage in COPD by facilitating the removal of damaged mitochondria, thereby contributing to the maintenance of normal cellular function and survival	Mitochondrial dysfunction: In COPD, disruptions in the process of mitophagy can result in mitochondrial dysfunction, potentially aggravating the pathological progression of pulmonary disease Effects of oxidative stress: The aberrations in mitophagy observed in COPD may be associated with oxidative stress, which could detrimentally impact airway epithelial cells	[17, 158, 159]
Skin diseases	Skin aging and skin cancer	Mitochondria are critical organelles impacted by aging processes induced by temporal factors and ultraviolet (UV) exposure in the skin, with phenotypic alterations arising directly from mitochondrial dysfunction. Furthermore, mtDNA deletions and other abnormalities are commonly observed in photoaged skin and regions affected by skin cancer	Mitochondria-dependent epidermal differentiation involves mitochondrial respiration generating ATP to fulfill the substantial energy demands of metabolically active cutaneous cells, thereby facilitating the perpetual self-renewal of the normal epidermis	Skin aging: a sustained decline in mitochondrial function, an increase in ROS production, loss of mitochondrial membrane potential (MMP), followed by an increase in mitophagy and apoptosis. This may accelerate the skin aging process, slowing down skin inflammation and wound healing	[160]
Eye diseases	Age-related macular degeneration	In the initial stages of age-related macular degeneration (AMD), the diminution of mitophagy and the compromised antioxidant signaling of nuclear factor E2-related factor 2 (NFE2L2) in retinal pigment epithelial cells may trigger epithelial–mesenchymal transition, which possesses anti-apoptotic characteristics, thereby influencing cellular survival and function	Mitophagy plays a crucial role in sustaining retinal metabolism and homeostasis by selectively degrading damaged mitochondria, thereby ensuring a healthy mitochondrial pool. This process is essential for the maintenance of metabolic reprogramming and the differentiation of retinal ganglion cells	Association with dry AMD: In a model of dry AMD, impaired mitophagy within retinal pigment epithelial cells is linked to the pathogenesis of AMD	[161, 162]
	Glaucoma	Elevated intraocular pressure is associated with an upregulation of mitophagy, which contributes to the progressive degeneration of retinal ganglion cells (RGCs), potentially resulting in irreversible blindness	Development of a treatment strategy: Enhancing Parkin expression or inhibiting uncoupling protein 2 can partially restore the autophagic activity of retinal ganglion cells under conditions of elevated intraocular pressure, thereby offering effective protection to RGCs in the context of glaucoma	Glaucoma-related neurodegeneration may be exacerbated by abnormalities in mitochondrial autophagy, which can intensify neuronal damage	[163, 164]
	Dry eye	The hypertonic tear environment induces mitochondrial oxidative damage and disrupts energy metabolism in human corneal epithelial cells (HCECs). This condition activates AMPK, triggering mitochondrial fission and mitophagy. Consequently, a detrimental cycle ensues, characterized by elevated ROS levels and exacerbated mitochondrial dysfunction	In response to oxidative stress, the upregulation of mitophagy facilitates the removal of damaged mitochondria, thereby diminishing the production of ROS and exerting a protective effect on cellular integrity	Exacerbation of oxidative damage and inflammation: In the context of dry eye disease, the dysregulated activation of mitophagy may intensify oxidative damage and inflammatory responses within corneal cells	[165]

To investigate the regulatory mechanisms of mitophagy, it has been identified that, beyond the classical receptors BNIP3 and FUNDC1, a range of autophagy receptors, including BNIP3L/NIX, BCL2L13, AMBRA1, and FKBP8, are localized within the outer mitochondrial membrane (OMM). These receptors possess the capability to directly recruit LC3/GABARAP proteins, thereby facilitating mitochondrial degradation independently of ubiquitin signaling [50, 166, 167]. Conversely, the inner mitochondrial membrane (IMM) typically remains impermeable; however, during ischemic injury, characterized by simultaneous nutrient and oxygen deprivation, the mitochondrial permeability transition pore (mPTP) opens nonselectively. This opening results in the uncoupling of oxidative phosphorylation, ATP hydrolysis, and the accumulation of intramitochondrial inorganic phosphate [31]. CL plays a critical role in this regulatory process, as most CL-mediated mitochondrial autophagy receptors contain LC3 interaction regions (LIRs), which enable them to bind tightly to LC3/GABARAP proteins [168]. This interaction facilitates the induction of mitophagy under cellular stress conditions through the association of CL with LC3 [50]. Moreover, lipids such as ceramides may serve as signaling molecules or receptors for compromised mitochondria [169]. Hypoxia and nutrient deprivation are potent inducers of autophagy; under anoxic conditions, cells activate the autophagic pathway to degrade and recycle intracellular components to maintain energy homeostasis and ensure survival [170, 171]. Autophagy is significantly upregulated in response to nutrient deprivation, particularly in the context of energy depletion due to deficiencies in amino acids and glucose [12, 72]. This process constitutes a cellular strategy to acquire essential nutrients and support survival [170]. Additional regulatory mechanisms include the dysregulation of lysosomal clearance, the involvement of NLRP3 inflammasomes in mediating pyroptosis, the role of iron metabolism-related proteins in influencing ferroptosis, and the regulation of mitochondrial damage by BCL-2 family proteins [172–174]. In recent years, ERS has also garnered significant attention as a novel regulatory pathway of apoptosis [175]. ERS is implicated in a wide range of physiological and pathological processes, including protein folding, intracellular Ca^2+^ storage, oxidative stress, hypoxia, ischemia, and lipid metabolism disorders, and is intricately associated with myocardial IRI [29]. Although ERS is essential for cellular survival, its prolonged activation can lead to apoptosis [7]. The myocardial damage resulting from the accumulation of unfolded proteins during ERS can further exacerbate ERS, thereby altering the metabolic state of cardiomyocytes and causing more severe injury [176]. In the context of MIRI, ERS levels increase, and the attenuation of ERS has been demonstrated to alleviate the effects of MIRI [29, 87, 100]. However, it is crucial to recognize that not all ERS responses are harmful. For example, the ERS transcription factor ATF6 has been shown to provide cardiomyocyte protection against ischemia–reperfusion injury [7]. Collectively, these mechanisms contribute to the impaired clearance of damaged proteins and organelles within cells, leading to their intracellular accumulation. This accumulation subsequently triggers apoptosis, necrosis, and other forms of cell death, thereby exacerbating myocardial damage [20]. Stress granules, which are membraneless organelles located within the cytoplasm, are formed in response to a variety of environmental stressors, such as elevated temperatures, oxidative stress, and viral infections [177]. These granules consist of messenger ribonucleoprotein complexes (mRNPs), including stalled mRNA, RNA-binding proteins (RBPs), translation initiation factors, and various other proteins [78]. Recent studies have highlighted the critical role of ubiquitination in regulating the dynamics of stress granules, particularly concerning their assembly, disassembly, and degradation processes [81, 177, 178]. The regulation of stress granule dynamics is significantly influenced by PTMs, including phosphorylation and methylation [178]. Different stressors induce distinct patterns of ubiquitination within the stress granule proteome; for instance, heat shock results in substantial ubiquitination of stress granule components, whereas arsenite, a common inducer of stress granules, does not [81].

We propose the novel use of autophagy regulation as a therapeutic strategy to mitigate organ damage and systemic effects resulting from I/R injury. To accomplish this, it is imperative to explore the molecular mechanisms underlying the dual role of autophagy and to develop pharmacological agents that are both safe and efficacious, ensuring their timely and precise administration. The challenge lies in identifying the critical factors, timing, and extent of autophagy activation, as well as synchronizing these interventions with the dynamics of I/R injury. Consequently, it is imperative to rigorously evaluate the impact of these regulatory strategies in preclinical models to ascertain their efficacy and potential side effects. The exploration of autophagy’s therapeutic potential is currently progressing, with each step offering new insights into the intricate balance between its protective and detrimental properties. Here, we offer a summary of the factors that can influence or regulate the process of autophagy, as delineated in Table 2.

**Table 2:** Autophagy-related influencing factors

Pharmacological interventions	Mechanism	Refs.
Rapamycin	Rapamycin serves as a highly effective and selective inhibitor of the mTOR signaling pathway, thereby promoting the process of autophagy	[91, 92]
Empagliflozin	The SGLT2 inhibitor, empagliflozin, confers cardioprotection by mitigating autophagic cell death in cardiomyocytes, which is induced by excessive autophagy. Furthermore, empagliflozin alleviates ischemia/reperfusion injury in cardiac microvasculature through the activation of the AMPKα1/ULK1/FUNDC1/mitochondrial autophagy signaling pathway	[21, 64, 166]
Calcium channel inhibitors	The elevation of intracellular Ca^2+^ levels and the consequent swelling of mitochondria expedite the autophagic process	[5, 8, 32, 176]
Carfilzomib (CFZ)	The administration of CFZ resulted in elevated levels of ubiquitinated BNIP3L and LC3B, thereby promoting autophagic activity	[30, 49, 50, 106]
Overactivation of the mTOR pathway	In colorectal cancer, the hyperactivation of the mTOR signaling pathway can suppress the initiation of autophagy, consequently facilitating the proliferation and survival of tumor cells	[60]
Genetic manipulation		
Circ-FoxO3	Circ-FoxO3 facilitates the modulation of autophagy or ATG through knockout or overexpression by inhibiting mTORC1	[59, 61]
ncRNAs	In the context of spinal cord ischemia–reperfusion injury (SCIRI), noncoding RNAs (ncRNAs) have the capacity to regulate apoptosis, inflammation, autophagy, and oxidative stress, thereby mitigating the effects of SCIRI	[100]
Beclin 1	Beclin 1 modulates autophagy via phosphorylation, while the pro-apoptotic kinase Mst1 can suppress autophagy by phosphorylating the BH3 domain of Beclin 1	[84, 85]
ATG gene	The protein encoded by the ATG gene is integral to the initiation and nucleation of autophagosomes. Specifically, ATG1, in conjunction with the ULK1/2 complex, and ATG13 are pivotal during the early stages of autophagosome formation. They form complexes with ATG14 and FIP200, which facilitate the initiation of autophagosomes. ATG proteins engage in intricate interactions to form various complexes, such as the ATG5–ATG12–ATG16L1 complex and the ATG8 (LC3) lipid system. Notably, the lipidated form of LC3, known as LC3-II, serves as a hallmark of autophagosome formation. These complexes are crucial for the expansion of the autophagosome membrane and its subsequent fusion	[20–22, 50, 65, 85, 94]
Environmental factors		
H_2_S	Low concentrations of hydrogen sulfide (H_2_S) have the potential to mitigate neuronal damage induced by cerebral ischemia–reperfusion (CIR)	[104]
BNIP3L	BNIP3 has been characterized as a pro-apoptotic protein, the induction of which has been demonstrated to enhance the insertion and activation of BAX (BCL2-associated X, apoptosis regulator) and BAK (BCL2 antagonist/killer 1) within the mitochondria	[49, 63, 110, 179]
ULK1	The phosphorylation of FUNDC1 by ULK1 has been demonstrated to activate FUNDC1-dependent mitophagy	[13, 21, 22, 65, 82, 85, 106]
PI3K	Phosphatidylinositol 3-kinase (PI3K)-activated protein kinase B (PKB) undergoes activation through direct phosphorylation of a pivotal component of the mTORC1. Phosphatidylinositol (3, 4, 5)-trisphosphate (PIP3) can activate PKB, which subsequently inhibits autophagy by phosphorylating and suppressing the activity of mTOR. In contrast to class I PI3K, class III PI3K plays a crucial role in the initiation of autophagy. Class III PI3K generates PI3P, a critical step in the formation of autophagosomes. Additionally, PIP3 functions as a second messenger, modulating other signaling molecules and kinases, thereby exerting an indirect regulatory effect on autophagy	[3, 22, 24, 28, 85, 94, 180]
PINK1-Parkin	The PINK1-PRKN/Parkin pathway facilitates the tagging of impaired mitochondria with ubiquitin chains, thereby initiating their selective autophagic degradation	[50, 51]
CL	Cardiolipin-mediated mitophagy triggers the initiation of mitophagy in response to cellular stress through the interaction between cardiolipin (CL) and microtubule-associated protein 1A/1B-light chain 3 (LC3)	[50, 172]
Ceramides	Ceramides have the capacity to induce autophagy through multiple mechanisms. They activate intracellular signaling pathways, including ERK and p38 MAPK, which play a crucial role in the initiation of autophagy	[169]
Hypoxia	Following hypoxic conditions, anaerobic glycolysis becomes the primary metabolic pathway, resulting in the accumulation of lactic acid and hydrogen ions, which in turn causes intracellular acidosis	[170, 171]
Nutritional deprivation	Under conditions of nutrient deprivation, cells initiate the degradation of their own components, including damaged proteins, organelles, and other biological macromolecules, to facilitate the synthesis of new molecules or to serve as an energy source. Furthermore, nutrient deprivation results in decreased levels of intracellular amino acids and growth factors, thereby inhibiting the mTOR signaling pathway and promoting the induction of autophagy	[12, 72]
NLRP3 inflammasomes	The activation of the NLRP3 inflammasome is induced by ROS and adenosine triphosphate (ATP), subsequently resulting in the secretion of the pro-inflammatory cytokines interleukin-1 beta (IL-1β) and interleukin-18 (IL-18), as well as the initiation of pyroptosis	[174]
Lysosomal clearance dysfunction	Lysosomal dysfunction can result in the accumulation of autophagic substrates within the cell, thereby impairing cellular function and potentially leading to cell death. Moreover, rupture or dysfunction of lysosomes may cause the release of their enzymes into the cytoplasm, which can initiate an inflammatory response and contribute to cellular demise	[3, 12]
Ferroptosis	Ferroptosis is characterized by the accumulation of lipid ROS originating from iron metabolism, with its primary features being mitochondrial condensation and increased bilayer membrane density	[9, 63, 72]
Nrf2	Nrf2 promotes the expression of antioxidant genes and, under nonstressed conditions, is sequestered in the cytoplasm through direct interaction with Keap1	[6, 181]
AMPK	Activated AMPK has the capability to mitigate oxidative stress by suppressing NADPH oxidase activity and enhancing antioxidant responses mediated by Nrf2	[182]

## The effects on organ damage and the whole body

Phagocytosis has extensive and significant implications for I/R-induced clinical organ injury and its systemic effects [60]. As previously discussed, autophagy represents a distinct form of cell death that operates independently of other apoptotic pathways or excessive autophagic processes [10].

**Fig. 4:**
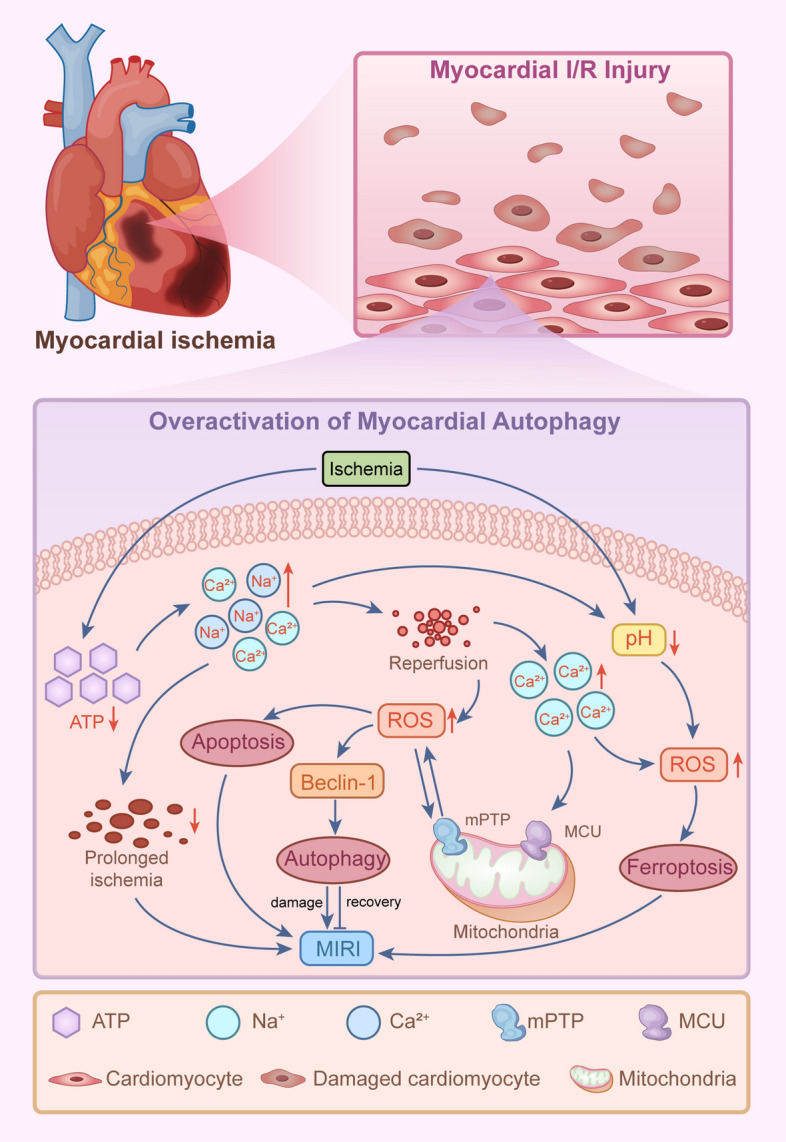
Deleterious effects of autophagy in MIRI. During ischemic conditions, cardiomyocytes shift to anaerobic metabolism, which leads to a reduction in ATP levels and an elevation in intracellular Na^+^ and Ca^2+^ concentrations. Upon reperfusion, there is an exacerbation of intracellular Ca^2+^ accumulation and ROS generation, culminating in MIRI. The principal apoptotic signaling pathways involved are the intrinsic pathway, mediated by mitochondrial mechanisms, and the extrinsic pathway, mediated by death receptors. During ischemia and the initial phase of reperfusion, cellular acidosis, disruption of homeostasis, and various other factors facilitate the enzymatic release of iron equivalents or iron ions. This process activates the Fenton reaction, resulting in elevated levels of ROS, ultimately inducing ferroptosis in cardiomyocytes. (Created using Adobe Illustrator)

Cardiovascular disease represents a significant global public health challenge, emerging as a leading cause of morbidity and mortality worldwide [183]. The investigation of autophagy within the context of cardiovascular diseases (CVDs) encompasses intricate vascular pathological processes that result in the impairment of vascular architecture and cardiac functionality, thereby imposing a substantial burden on global health systems and economic resources [184]. These conditions encompass atherosclerosis, hypertension, MIRI, myocardial infarction, myocardial hypertrophy, heart failure, and dilated cardiomyopathy [185, 186]. Adverse consequences of autophagy in MIRI are illustrated in Fig. 4.

Mortality rates associated with cardiovascular disease have decreased as a result of proactive preventive strategies and advancements in pharmacological treatments and medical technology [166]. Nonetheless, the overall burden of cardiovascular disease remains substantial, primarily owing to inadequate implementation of prevention guidelines, challenges in adhering to preventive measures, and the prevalent occurrence of risk factors for coronary heart disease, including lipid metabolism disorders, hypertension, and diabetes mellitus [107]. The issue of IRI in the context of myocardial infarction presents a significant challenge in cardiovascular medicine. The acute loss of myocardial tissue due to an ischemic event results in profound metabolic and ionic disturbances within the affected myocardium, culminating in cell death [187]. Even when blood flow is subsequently restored, the heart’s limited regenerative capacity poses a substantial obstacle to cell replacement [188]. The role of autophagy in cardiac function is complex and somewhat paradoxical. In conditions such as obesity and high-fat diet-induced cardiac dysfunction, autophagy plays a protective role by modulating cardiometabolism and safeguarding the heart against ischemia–reperfusion damage [30, 189, 190]. Conversely, cardiomyocytes are heavily reliant on oxygen for oxidative phosphorylation to sustain their function. Under hypoxic conditions, the reduction in mitochondrial ATP production leads to calcium overload, acidosis, and oxidative stress, all of which contribute to myocardial injury [191]. In the context of cardioprotective effects, pretreatment has been shown to enhance autophagic flux by activating AMPK and PI3K while inhibiting mTOR [192]. Additionally, it has been proposed that drug posttreatment (PPC), administered within minutes during the early stages of reperfusion, offers greater efficacy and flexibility compared with conditioning or ischemic conditions [193]. Several studies have indicated that fluctuations in oxygen levels, rather than sustained low oxygen levels, constitute the most detrimental factor in myocardial hypoxia–reperfusion injury. Therefore, it is imperative to regulate the reperfusion process in hypoxia–reperfusion injury to prevent significant myocardial damage caused by abrupt, extensive blood reperfusion [194].

Cerebral ischemic injury is a significant contributor to global morbidity and mortality, precipitating various central nervous system disorders, including AIS and chronic ischemic AD [111]. Research indicates that, during cerebral ischemia/reperfusion events, there is a reduction in ATP levels, which activates the intracellular energy sensor AMPK. This activation subsequently inhibits mTORC1, resulting in the dephosphorylation of ATG13 and ULK1. These molecular events facilitate the assembly of ULK1 complexes and expedite the initiation of autophagy [195, 196]. During the initial phase of reperfusion, autophagy plays a cytoprotective role by degrading damaged organelles and misfolded proteins, thereby releasing amino acids and nucleotides for recycling [197]. Conversely, prolonged activation of autophagy in the later stages of reperfusion may result in the excessive degradation of healthy organelles and proteins, culminating in autophagic cell death and secondary injury to histiocytes. This indicates that autophagy has a dual role in brain I/R injury [198].

HIRI represents a significant complication associated with hepatectomy and liver transplantation, profoundly affecting patient outcomes [199]. Research indicates that upregulated autophagy plays a crucial role in the restoration of liver function following I/R injury. From an energy metabolism perspective, autophagy contributes to the maintenance of metabolic homeostasis by facilitating the removal of damaged organelles and proteins, thereby recycling their constituent nutrients and supplying energy to cells [200]. During oxidative stress, autophagy plays a crucial role in mitigating cellular damage by eliminating deleterious substances, including ROS. This process helps to attenuate the detrimental effects of oxidative stress on cells. Furthermore, within the context of the inflammatory response, autophagy serves to inhibit the release of inflammatory cytokines, thereby reducing the inflammatory cascade and alleviating liver injury [27, 201]. Nonetheless, it is important to note that excessive autophagy can result in the degradation of normal organelles and proteins, which may impair cellular function and potentially exacerbate liver injury [202]. The regulatory mechanisms governing autophagy are intricate, and the interactions among various pathways render the role of autophagy in HIRI a subject of ongoing debate [201]. The AMPK/mTOR signaling pathway is unequivocally central to the regulation of autophagy and remains a prominent subject of contemporary research. Studies have demonstrated that the induction of autophagy correlates with reduced expression and activity of mTOR during HIRI. Furthermore, autophagy displays dual regulatory roles in the progression of HIRI, exerting a protective effect on cells during the early stages, while potentially contributing to adverse outcomes in prolonged ischemic conditions [202]. As previously discussed, the removal of damaged mitochondria is dependent on the selective autophagic process mediated by the PINK1/Parkin pathway, known as mitophagy [203]. The upregulation of PINK1 protein via this pathway has been demonstrated to trigger mitophagy, subsequently inhibiting the NLRP3 inflammatory pathway and mitigating HIRI [204].

Autophagy is an essential mechanism for the kidneys to sustain normal physiological functions, including the preservation of podocyte morphology and functionality [13, 61]. The targeted deletion of ATG5 or ATG7 in renal epithelial cells has been demonstrated to induce CKD in murine models, characterized by podocyte and tubular dysfunction, glomerular and tubulointerstitial damage, and progressive organ failure [205]. Moreover, the specific deletion of ATG5 in mouse podocytes facilitates the onset of age-dependent glomerulopathy, evidenced by the accumulation of oxidative and ubiquitinated proteins, heightened endoplasmic reticulum stress, podocyte loss, and proteinuria [61]. These studies have substantiated the critical role of autophagy in mitigating age-related glomerular disease and the deterioration of renal function [61]. Beyond glomerular disease, the damage and apoptosis of tubular epithelial cells represent a significant characteristic of AKI, which has the potential to progress to CKD if the injury is recurrent or inadequately repaired [206]. Cisplatin-induced tubular apoptosis has been demonstrated to decrease when autophagy inhibitors, such as 3-methyladenosine or bafilomycin, are used, or when Beclin-1 expression is downregulated. This is particularly relevant for chemotherapeutic agents such as cisplatin, which induce AKI through the promotion of autophagy [207]. Nonetheless, certain studies have indicated that the enhancement of autophagy by cisplatin may, in contrast, aggravate renal injury and apoptosis [208]. Various injuries, including renal I/R, sepsis, and exposure to nephrotoxins, can result in nutrient depletion and oxidative stress, which subsequently trigger the activation of autophagy [13, 14, 61]. Renal fibrosis is a characteristic feature of CKD, with transforming growth factor-β1 (TGF-β1) playing a pivotal role in its progression. TGF-β1 not only facilitates the activation of fibroblasts but also significantly contributes to the development of renal fibrosis [209, 210]. In the context of chronic kidney diseases, including diabetic nephropathy, primary nephrotic syndrome, immunoglobulin A nephropathy, and doxorubicin-induced nephropathy, autophagy may be activated as an intrinsic protective mechanism within renal tubular epithelial cells and podocytes. The extent of autophagic disruption is associated with the severity of chronic kidney disease exacerbation [211–213].

Furthermore, autophagy is essential for preserving endothelial cell function and vascular integrity during pulmonary I/R injury [185]. Autophagy, a crucial cellular degradation and recycling mechanism, plays a vital role in preserving damaged mitochondria and preventing the release of cytotoxic substances, thereby sustaining mitochondrial function and cellular viability. The regulation of autophagy within endothelial cells influences the equilibrium between pro-survival and pro-apoptotic signaling pathways, potentially altering cellular fate under ischemic conditions [214]. Furthermore, autophagy inhibits apoptosis, maintains intracellular homeostasis, and safeguards blood vessels from damage by modulating angiogenesis [25]. For instance, P66shc facilitates the removal of damaged mitochondria through the promotion of mitophagy, thereby contributing to the maintenance of mitochondrial function and vascular integrity in endothelial cells [185]. Dysfunction of pulmonary endothelial cells (ECs) is a key characteristic of pulmonary I/R injury, leading to excessive fluid accumulation in the lungs (i.e., edema), reduced efficiency of gas exchange, and diminished lung elasticity [215]. The occurrence of I/R injury following lung transplantation is unavoidable and can result in the initial dysfunction of the transplanted organ. This condition contributes to heightened morbidity and mortality among postoperative patients and may also precipitate immune rejection, a critical determinant of postoperative mortality in recipients [216]. Dysfunction in autophagy can result in vascular injury and disease. Consequently, strategies aimed at modulating autophagy, including the application of autophagy inducers, inhibitors, or gene therapy, are anticipated to enhance vascular integrity and offer therapeutic benefits for vascular diseases [84, 92]. Moreover, interventions targeting autophagy-related signaling pathways, as well as the use of natural products and lifestyle modifications, present additional avenues for modulating autophagy to improve vascular health [217, 218].

The impact of autophagy on the immune system’s inflammatory response is significant: autophagy has the potential to attenuate inflammation by degrading pro-inflammatory cytokines and modulating antigen presentation to immune cells [27]. Nevertheless, an imbalance in this regulatory mechanism can lead to exacerbated inflammation, potentially initiating a cascade of events culminating in multi-organ dysfunction syndrome [12]. Nod-like receptors (NLRs), including NOD1 and NOD2 signaling pathways, play a crucial role in immune defense by inducing autophagy and suppressing inflammatory responses. Additionally, autophagy modulates inflammatory pathways in macrophages, such as the NF-κB pathway, the RIG-I/STING pathway, and the inflammasome pathway [60, 201]. Disruptions in autophagy can result in heightened inflammatory responses, including conditions such as IBD, systemic lupus erythematosus (SLE), and arthritis. For instance, a deficiency in ATG7 is associated with elevated levels of IL-1β and pyroptosis, while a deficiency in ATG5 increases vulnerability to *Mycobacterium tuberculosis* [46, 201]. Consequently, investigating the interplay between autophagy and macrophage function is crucial for elucidating the mechanisms underlying inflammatory responses and for devising novel therapeutic strategies for inflammatory diseases [27]. Comprehending the organ-specific and systemic functions of autophagy in I/R injury is essential for the advancement of targeted therapeutic strategies. It is imperative to sustain a delicate equilibrium to avert dysregulation in the role of autophagy within these processes. The dual roles of autophagy in the IRI process of different organs are listed in Table 3.

**Table 3:** The dual role of autophagy in ischemia–reperfusion injury in different organs

Organ	Protective effects	Harmful effects	Influencing factors	Refs.
Heart	Autophagy serves as a critical cellular mechanism for mitigating oxidative stress and the accumulation of toxic substances by facilitating the removal of damaged organelles and proteins, thereby preserving intracellular homeostasis. Furthermore, moderate activation of autophagy is pivotal during the ischemia–reperfusion process in cardiomyocytes, contributing to the maintenance of cellular energy metabolism balance. Additionally, autophagy attenuates the release of inflammatory mediators through the clearance of damaged organelles, thus reducing further damage to cardiomyocytes and inhibiting the onset of inflammatory responses	In the context of myocardial IRI, excessive activation of autophagy may result in the degradation of critical organelles and proteins within cells, thereby compromising cellular structure and function, ultimately culminating in cell death. Autophagy is intricately linked to apoptosis, and during myocardial IRI, there is typically a reduction in mTOR activity accompanied by an upregulation of Beclin1 expression. This dynamic fosters the activation of both autophagy and apoptosis, thereby exacerbating cardiomyocyte damage	The role of mitochondrial autophagy in cardiac IRI is nuanced. A moderate level of mitochondrial autophagy facilitates the removal of damaged mitochondria, thereby mitigating mitochondrial damage and conferring protection to cardiomyocytes. Conversely, excessive activation of mitophagy can result in a substantial depletion of mitochondria, adversely impacting the energy metabolism of cardiomyocytes	[219–221]
Kidney	In the context of mild IRI, autophagy plays a protective role by mitigating the release of inflammatory cytokines through the removal of damaged organelles and proteins, thereby attenuating the inflammatory response and subsequent damage to renal cells. Within a specific threshold, autophagy serves to protect renal tubular cells by inhibiting apoptosis	In cases of severe IRI, excessive activation of autophagy can compromise essential organelles and proteins within kidney cells, potentially facilitating apoptosis and exacerbating cellular injury. This overactivation may undermine the structural integrity of kidney cells and promote apoptosis through various mechanisms, leading to an intensification of apoptosis that further exacerbates cellular damage and significantly impairs renal function	Mitophagy plays a critical role in the selective removal of damaged mitochondria, primarily through mechanisms such as the PINK1/Parkin pathway. This process not only reduces the production of reactive oxygen species (ROS) but also facilitates mitochondrial degradation via the autophagy pathway, thereby mitigating kidney damage. Under conditions of endoplasmic reticulum stress, the activation of the PERK and IRE1 pathways enhances autophagosome formation, aiding in the clearance of misfolded proteins and reducing cellular damage associated with endoplasmic reticulum stress	[138, 222, 223]
Liver	During ischemia–reperfusion, the energy supply to hepatocytes is significantly constrained. In such scenarios, moderate autophagy is crucial for maintaining cell viability by degrading intracellular components to provide energy. Furthermore, autophagy contributes to the removal of damaged mitochondria and proteins, thereby reducing oxidative stress and cellular damage, ultimately exerting a protective effect on liver cells	In instances of severe IRI, excessive activation of autophagy can result in the degradation of critical intracellular organelles and proteins, thereby compromising the structural and functional integrity of the cell and ultimately inducing cell death. This phenomenon holds significant importance in the pathological mechanisms underlying cellular injury, indicating that therapeutic approaches for IRI should focus on the precise modulation of autophagy to prevent further cellular damage due to its overactivation	Beyond the regulation of endoplasmic reticulum stress and mitophagy, microRNAs (miRNAs) are also pivotal in modulating autophagy in hepatic IRI. For instance, miR-17 exacerbates the pathological damage associated with IRI by enhancing autophagic activity. This suggests a potential regulatory role in the pathogenesis and progression of hepatic IRI, highlighting miR-17 as a prospective therapeutic target for the treatment of hepatic IRI in the future	[224, 225]
Brain	Physiological levels of autophagy play a protective role in neuronal cells by inhibiting apoptotic processes. For instance, pharmacological agents that stimulate autophagy, such as spermine, enhance autophagic activity through the activation of the AMPK/mTOR/ULK1 signaling pathway, thereby mitigating inflammation and apoptosis. Additionally, autophagy facilitates the recovery of neural tissue following ischemic events by modulating microglial phenotypic changes and engaging the NF-κB pathway	Following ischemic brain injury, excessive activation of autophagy can impair neuronal function, resulting in neurological deficits such as impaired learning and memory, as well as motor dysfunction	Consequently, modulating autophagy activity holds promise for mitigating neurological deficits post-ischemic brain injury and offers novel insights and targets for clinical interventions	[226–228]
Eye	In the context of retinal IRI (RIRI), autophagy plays a crucial role in mitigating oxidative stress damage by facilitating the removal of damaged mitochondria and decreasing the production of reactive oxygen species (ROS). Research indicates that modulating autophagic activity can significantly enhance the survival rate of retinal ganglion cells following ischemia–reperfusion. This suggests that autophagy not only alleviates oxidative stress through the clearance of damaged organelles but may also confer a protective effect by modulating inflammatory responses and apoptosis pathways	In cases of severe IRI in the eye, autophagy may be markedly activated, potentially leading to retinal dysfunction. For instance, 3-methyladenine (3-MA), a well-established autophagy inhibitor, has been shown to mitigate retinal IRI by curbing autophagy overactivation. Thus, 3-MA presents potential therapeutic value in treating retinal IRI, offering a viable strategy for inhibiting excessive autophagy	Inhibition of autophagic activity in rat models has been shown to reduce LC3-II levels, thereby diminishing the neuroprotective effects of ischemic posttreatment and exacerbating histological damage to the omentum. These findings imply that autophagy exhibits a complex dual role in retinal IRI, and precise regulation of autophagic activity is pivotal for achieving neuroprotection in the treatment of this condition	[229, 230]

Future therapeutic approaches must meticulously modulate autophagic activity to optimize its protective benefits while mitigating the potential risk of harm [12]. An important consideration is that autophagy inhibitors impact various stages of the autophagic process, resulting in distinct therapeutic outcomes. During the initiation phase of autophagy, inhibitors such as 3-methyladenosine, wortmannin, and LY294002 can impede the onset of autophagy. This inhibition leads to a downregulation in the expression of autophagy-related proteins, specifically LC3-II and Beclin-1, consequently diminishing autophagic flux. During the fusion phase of autophagy, inhibitors such as pafimycin A1 and chloroquine impede the fusion of lysosomes with autophagosomes, thereby obstructing the degradation of autophagic contents. This results in a reduction of autophagic activity, yet concurrently leads to an upregulation in the expression of autophagy-related proteins and an enhancement in autophagic flux [199]. This therapeutic approach within precision medicine necessitates an in-depth and comprehensive understanding of the cellular context, as well as the interactions between autophagy and other cell death mechanisms. Such understanding is essential to accurately discern the intricate balance between autophagy and cellular rescue or destruction following I/R injury. This knowledge may offer novel insights for the development of innovative treatments for I/R injury.

## Conclusions

The dual role of autophagy in ischemia–reperfusion (I/R) injury presents both a challenge and an opportunity in therapeutic strategies. As a double-edged sword, autophagy can either protect or harm cells, depending on the context and extent of activation. This duality demands a nuanced understanding of its mechanisms and regulatory pathways. During the ischemic phase, autophagy acts as a protective mechanism by clearing dysfunctional organelles and misfolded proteins, thus maintaining cellular integrity. This is particularly important in organs such as the brain and heart, where rapid response to damage is critical. Autophagy recycles cellular components to restore energy balance, thereby facilitating recovery upon reperfusion. Conversely, excessive autophagy during reperfusion can exacerbate tissue damage. Overactivation might lead to autophagic cell death or an imbalance in cellular homeostasis, as seen in cases where it contributes to mitochondrial fragmentation and ATP depletion. Understanding the thresholds of protective versus detrimental autophagy is crucial, especially in therapeutic contexts such as cancer treatment or organ transplants, where modulation of autophagy could shift outcomes significantly. The therapeutic potential lies in precisely regulating autophagy to harness its protective benefits while minimizing harmful effects. This requires a sophisticated approach, potentially involving the use of autophagy modulators at specific stages of the process. For instance, targeting the AMPK/mTOR pathway could effectively modulate autophagy in liver and kidney injuries, improving outcomes in organ transplantation and chronic disease management. The future of I/R injury treatment could be revolutionized by therapies that finely tune autophagic responses. Investigating molecular targets such as the PINK1/Parkin pathway in mitochondrial autophagy could provide insights into reducing oxidative stress and inflammation. Additionally, understanding the interplay between autophagy and other cell death pathways could lead to more effective and personalized therapeutic strategies.

In conclusion, autophagy’s dual role in I/R injury underscores the need for targeted therapeutic approaches that leverage its protective aspects while controlling its potential to cause harm. As research advances, the potential for autophagy-based therapies in improving outcomes for diseases involving I/R injury appears promising, provided that we achieve a deeper comprehension of its regulatory mechanisms and their clinical implications.

## Data Availability

Data sharing is not applicable to this article as no datasets were generated or analyzed during the current study. All information is derived from publicly available articles and datasets.

## Abbreviations

Aβ: β-Amyloid
ACD: Autophagic cell death
AD: Alzheimer’s disease
ADCD: Autophagy-dependent cell death
AIS: Acute ischemic stroke
AKI: Acute kidney injury
ALI: Acute lung injury
ALS: Amyotrophic lateral sclerosis
AMBRA1: Activating molecules of the autophagy regulator 1
AMCD: Autophagy-mediated cell death
AMD: Age-related macular degeneration
AMP: Adenosine monophosphate
AMPK: AMP-activated protein kinase
ATGs: Autophagy-related genes
ATP: Adenosine triphosphate
BAK: BCL-2 antagonist/killer 1
BAX: BCL-2-associated X
BBB: Blood–brain barrier
BCL-2: B-cell lymphoma 2
BIM: BCL-2 interacting mediator of cell death
CIRI: Cerebral ischemia–reperfusion injury
circ-FoxO3: Circular RNA FoxO3
cGAS: Cyclic GMP-AMP synthetase
CKD: Chronic kidney disease
CL: Cardiolipin
CMA: Chaperone-mediated autophagy
COPD: Chronic obstructive pulmonary disease
CVDs: Cardiovascular diseases
DAMPs: Damage-associated molecular patterns
DR: Death receptor
Drp1: Dynein-associated protein 1
ECs: Endothelial cells
ERS: Endoplasmic reticulum stress
FasL: Fas ligand
FasR: Fas receptor
GABARAP: Gamma-aminobutyric acid receptor-associated protein
H_2_S: Hydrogen sulfide
HF: Heart failure
HIRI: Hepatic ischemia–reperfusion injury
HSC: Heat shock cognate
IBD: Inflammatory bowel disease
IMM: Inner mitochondrial membrane
I/R: Ischemia–reperfusion
Keap1: Kelch-like ECH-associated protein 1
LAMP: Lysosome-associated membrane protein
LC3: Microtubule-associated protein 1 light chain 3
LIRs: LC3 interaction regions
LRRK2: Leucine-rich repeat kinase 2
MAPK: Mitogen-activated protein kinase
MIRI: Myocardial ischemia–reperfusion injury
miRNA: MicroRNA
MODS: Multiple organ dysfunction syndrome
mPTP: Mitochondrial permeability transition pore
mRNPs: Messenger ribonucleoprotein complexes
Mst1: Mammalian sterile 20-like kinase 1
mtDNA: Mitochondrial DNA
mTOR: Mammalian target of rapamycin
mTORC1: Mammalian target of rapamycin complex 1
MUL1: Mitochondrial E3 ubiquitin ligase
NAFLD: Nonalcoholic fatty liver disease
NBR1: Neighbor of BRCA1 gene 1
ncRNAs: Non-coding RNAs
NDP52: Nuclear dot protein 52
NLRP3: Nucleotide-binding domain and leucine-rich repeat containing protein 3
NLRs: Nod-like receptors
Nrf2: Nuclear factor erythroid 2-related factor
OMM: Outer mitochondrial membrane
Opa1: Optic atrophy 1
PI3K: Phosphatidylinositol 3-kinase
PI3P: Phosphatidylinositol 3-phosphate
PIP3: Phosphatidylinositol (3,4,5)-trisphosphate
PKB: Protein kinase B
pTau: Tau protein
PTMs: Posttranslational modifications
RBPs: RNA-binding proteins
RGCs: Retinal ganglion cells
ROS: Reactive oxygen species
SCIRI: Spinal cord ischemia–reperfusion injury
SGLT2: Sodium-glucose co-transporter 2
SIRS: Systemic inflammatory response syndrome
SLE: Systemic lupus erythematosus
SNARE: Soluble *N*-ethylmaleimide-sensitive factor attachment protein receptors
SOD: Superoxide dismutase
SQSTM1: Sequestosome 1
TGF-β1: Transforming growth factor-β1
TNF-α: Tumor necrosis factor-alpha
TNFR: Tumor necrosis factor receptor
TRAIL: TNF-related apoptosis-inducing ligand
TXNIP: Thioredoxin-interacting protein
UBAs: Ubiquitin-associated domains
UBDs: Ubiquitin binding domains
ULK: Unc-51 like autophagy activating kinase
UPS: Ubiquitin–proteasome system
Vps: Vacuolar protein sorting
